# Tregs Modulate Lymphocyte Proliferation, Activation, and Resident-Memory T-Cell Accumulation within the Brain during MCMV Infection

**DOI:** 10.1371/journal.pone.0145457

**Published:** 2015-12-31

**Authors:** Sujata Prasad, Shuxian Hu, Wen S. Sheng, Amar Singh, James R. Lokensgard

**Affiliations:** Neuroimmunology Laboratory, Center for Infectious Diseases and Microbiology Translational Research, Department of Medicine, University of Minnesota, Minneapolis, Minnesota, 55455, United States of America; University of Iowa, UNITED STATES

## Abstract

Accumulation and retention of regulatory T-cells (Tregs) has been reported within post viral-encephalitic brains, however, the full extent to which these cells modulate neuroinflammation is yet to be elucidated. Here, we used Foxp3-DTR (diphtheria toxin receptor) knock-in transgenic mice, which upon administration of low dose diphtheria toxin (DTx) results in specific deletion of Tregs. We investigated the proliferation status of various immune cell subtypes within inflamed central nervous system (CNS) tissue. Depletion of Tregs resulted in increased proliferation of both CD8^+^ and CD4^+^ T-cell subsets within the brain at 14 d post infection (dpi) when compared to Treg-sufficient animals. At 30 dpi, while proliferation of CD8^+^ T-cells was controlled within brains of both Treg-depleted and undepleted mice, proliferation of CD4^+^ T-cells remained significantly enhanced with DTx-treatment. Previous studies have demonstrated that Treg numbers within the brain rebound following DTx treatment to even higher numbers than in untreated animals. Despite this rebound, CD8^+^ and CD4^+^ T-cells proliferated at a higher rate when compared to that of Treg-sufficient mice, thus maintaining sustained neuroinflammation. Furthermore, at 30 dpi we found the majority of CD8^+^ T-cells were CD127^hi^ KLRG1^-^ indicating that the cells were long lived memory precursor cells. These cells showed marked elevation of CD103 expression, a marker of tissue resident-memory T-cells (T_RM_) in the CNS, in untreated animals when compared to DTx-treated animals suggesting that generation of T_RM_ is impaired upon Treg depletion. Moreover, the effector function of T_RM_ as indicated by granzyme B production in response to peptide re-stimulation was found to be more potent in Treg-sufficient animals. Taken together, our findings demonstrate that Tregs limit neuroinflammatory responses to viral infection by controlling cell proliferation and may direct a larger proportion of lymphocytes within the brain to be maintained as T_RM_ cells.

## Introduction

Regulatory T-cells (Tregs) are well-known to play crucial roles in suppression of immune responses during infection, as well as autoimmunity, and several recent studies describe their role in antiviral immunity [1, 2]. The role of these cells varies from controlling over whelming inflammation to local modulation of immune cells at sites of infection, thus executing effective immune responses [3]. Infection of the central nervous system (CNS) of mice with murine cytomegalovirus (MCMV) is characterized by long-term neuroinflammation which persists even in the absence of detectable levels of viral antigen. Neuroinflammation plays an important role in the pathogenesis of MCMV brain infection, with peripheral immune cell infiltration of the brain, activation of resident microglia, and production of proinflammatory cytokines [4, 5]. In addition, the CNS reservoir of latent and persisting viruses represents a potential source of clinically important virus [6]. Evidence from recent reports demonstrates that Treg cells also accumulate within the brains of mice upon viral infection and play distinct roles in immune modulation [7, 8]. Thus, proper Treg control over neuroimmune responses may be critical, particularly in sensitive tissues like the CNS, where heightened immune responses could evoke irreparable damage. Several previous studies have reported that the presence of Tregs within the brain has a significant impact on neuroimmune responses. They serve to limit tissue damage in many chronic infections. It has been shown that depletion of Tregs leads to more robust generation of effector T-cells, as well as short lived effector cells, in response to viral infection [7, 9].

Our laboratory has been actively investigating various inflammatory mechanisms following viral brain infection. Using our MCMV experimental brain infection model, we have previously reported that CD8^+^ T-cells persisted within the infected brain even when there is no substantial detectable viral gene product. In addition, long-term microglial cell activation driven by IFN-γ production by these T-cells has been demonstrated [10]. Through the use of specific Treg ablation, via administration of diphtheria toxin, we recently reported increased numbers of CD8^+^ and CD4^+^ T-cells within the brain of infected, Treg-deficient animals when compared to Treg-sufficient mice. In addition to this marked exacerbation of encephalitis, elevated expression of MHC class II, as well as PD-L1, on resident microglia was observed. Sustained microgliosis as well as increased glial fibrillary acidic protein (GFAP) expression on astrocytes were also seen in DTx-treated, infected animals. These findings indicate that neuroinflammation occurs concomitantly with accumulation and retention of immunosuppressive Tregs and demonstrate the presence of a heightened proinflammatory state following their ablation [8]. Thus, Tregs promote appropriate neuroimmune responses to viral brain infection by striking a balance between pathogen elimination and immune-mediated pathology.

Previous findings have demonstrated a role for cytotoxic T- lymphocytes in antiviral and antitumor immune responses, which is mediated by specialized secretory lysosomes containing granzyme B and perforin leading to specific target cell death [11, 12]. Upon receiving adequate costimulatory and inflammatory signaling during viral infection, naive CD8^+^ T-cells rapidly proliferate and differentiate into effector cells. After mediating pathogen clearance, the majority of effector cells die via apoptosis and are referred as short-lived effector cells. In contrast, small subsets of lymphocytes survive to form a pool of long-lived memory cells. Tremendous advancement has been made over the last ten years in identifying distinctive surface markers that are preferentially associated with either short-lived effector cell or long-lived memory cells [13, 14]. Being a characteristic feature of adaptive immunity, long-lived memory cells bear unique properties which permit vigorous, rapid, and specific responses upon Ag re-exposure. One type of memory cells is T-cells that express the α-chain (CD103) of the integrin α_E_β_7,_ termed tissue-resident memory T cells (T_RM_), [15, 16]. Generation of the T_RM_ subset is an especially important outcome of viral infection in immune privileged organs such as gut, brain, skin [17, 18]. Even though the literature explains much about the generation of these T_RM_ cells, greater understanding of their role within the CNS is needed. Thus, in this study we investigated the contribution of Tregs to modulating proliferation and activation of brain-infiltrating T lymphocytes, characterized their role in the transition of effector- memory CD8^+^ T-cells within the brain from acute through chronic phases, and examined their effect on the generation of T_RM_ cells in response to viral brain infection.

## Materials and Methods

### Ethical statement

This study was carried out in strict accordance with recommendations in the Guide for the Care and Use of Laboratory Animals of the National Institutes of Health. The protocol was approved by the Institutional Animal Care and Use Committee (Protocol Number: 1402-31338A) of the University of Minnesota. All surgery was performed under Ketamine /Xylazine anesthesia and all efforts were made to minimize suffering.

### Virus and animals

RM461, a MCMV expressing Escherichia coli β-galactosidase under the control of the human ie1/ie2 promoter/enhancer [19] was kindly provided by Edward S. Mocarski. The virus was maintained by passage in weanling female Balb/c mice. Salivary gland-passed virus was then grown in NIH 3T3 cells for 2 passages, which minimized any carry-over of salivary gland tissue. Infected 3T3 cultures were harvested at 80% to 100% cytopathic effect and subjected to three freeze–thaw cycles. Cellular debris was removed by centrifugation (1000Xg) at 4°C, and the virus was pelleted through a 35% sucrose cushion (in Tris-buffered saline [50 mMTris–HCl, 150 mMNaCl, pH 7.4]) at 23,000Xg for 2 h at 4°C. The pellet was suspended in Tris buffered saline containing 10% heat-inactivated fetal bovine serum (FBS). Viral stock titers were determined on 3T3 cells as 50% tissue culture infective doses (TCID_50_) per milliliter. Foxp3-GFP [20], as well as Foxp3-DTR [21], transgenic mice, each backcrossed over 15 generations onto the C57B/6 background [22], were kindly provided by Sing Sing Way (Cincinnati Children’s Hospital, Cincinnati, OH).

### Intracerebroventricular infection of mice

Infection of mice with MCMV was performed as previously described [23]. In brief, female mice (6–8 week old) were anesthetized using a combination of Ketamine and Xylazine (100 mg and 10 mg/kg body weight, respectively) and immobilized on a small animal stereotactic instrument equipped with a Cunningham mouse adapter (Stoelting Co., Wood Dale, IL). The skin and underlying connective tissue were reflected to expose reference sutures (sagittal and coronal) on the skull. The sagittal plane was adjusted such that bregma and lambda were positioned at the same coordinates on the vertical plane. Virulent, salivary gland-passaged MCMV RM461 (1 × 10^5^ TCID_50_ units in 10 μl), was injected into the right lateral ventricle at 0.9 mm lateral, 0.5 mm caudal, and 3.0 mm ventral to bregma using a Hamilton syringe (10 μl) fitted to a 27 G needle. The injection was delivered over a period of 3–5 min. The opening in the skull was sealed with bone wax and the skin was closed using 4–0 silk sutures with a FS-2 needle (Ethicon, Somerville NJ).

### Regulatory T-cell ablation

To address the effects of acute Treg depletion on chronic neuroinflammation following viral encephalitis, transgenic Foxp3-DTR mice were treated with diphtheria toxin (DTx, Sigma; CAT#: D0564). Stock DTx was diluted to a concentration of either 0.5 μg/100μl or 0.1 μg/100μl. On the day prior to intracerebroventricular (ICV) viral infection, mice were given intraperitoneal injections of DTx (0.5 μg/100μl). Subsequent doses of 0.1μg/100μl were given at 1 and 4 dpi to maintain Treg depletion throughout the acute phase of viral infection.

### Brain leukocyte isolation and flow cytometry analysis

Mononuclear cells were isolated from the brains of MCMV-infected transgenic Foxp3-DTR mice, with or without DTx treatment, using a previously described procedure with minor modifications [24–26]. In brief, whole brain tissues were harvested, pooled (n = 4–6 animals/group/ experiment), and minced finely using a scalpel in RPMI 1640 (2 g/L D-glucose and 10 mM HEPES) and digested in 0.0625% trypsin (in Ca/Mg-free HBSS) at room temperature for 20 min. Single cell preparations of infected brains were suspended in 30% Percoll and banded on a 70% Percoll cushion at 900 × g at 15°C. Brain leukocytes obtained from the 30–70% Percoll interface were collected.

Following preparation of single cell suspensions, cells were treated with Fc block (anti-CD32/CD16 in the form of 2.4G2 hybridoma culture supernatant with 2% normal rat and 2% normal mouse serum) to inhibit nonspecific Ab binding. Cells were then counted using the trypan blue dye exclusion method, and 1 x 10^6^ cells were subsequently stained with anti-mouse immune cell surface markers for 15 min at 4°C (anti-CD45-PE-Cy5, anti-CD8-PE-Cy7, anti-CD4-e-F 450, anti-CD11b-AF700, anti-ICOSPE-Cy5, anti-KLRG1PE-Cy7 and anti-103-1-PE (eBioscience, San Diego CA). Analysis by flow cytometry was performed. Control isotype Abs were used for all fluorochrome combinations to assess nonspecific Ab binding. Live leukocytes were gated using forward scatter and side scatter parameters on a BD FACSCanto flow cytometer (BD Biosciences, San Jose CA). Data were analyzed using FlowJo software (FlowJo, Ashland, OR).

### Intracellular cytokine staining

To determine the synthesis of intracellular cytokine production, brain mononuclear cells were stimulated either with polyclonal stimulation using anti-CD3/CD28 or an MCMV-specific M45 peptide (HGIRNASFI), which has previously been identified as a T-cell epitope [27, 28]. CNS lymphocytes were resuspended at 2 x10^6^ cells/well in RPMI complete medium supplemented with 10% FBS, 2μg/ml peptide and 1μl/ml Golgistop (BD Pharmingen) and incubated for 5h at 37°C (Horner et.al.2001). Peptide was omitted in negative control samples. Cells were surface stained prior to fixation/permiabilization using cytofix/cytosperm kit (eBioscience). Cells were then stained for IFN-γ eF450 (eBioscience) and TNF-α BV510 (Biolegend), as recommended by manufacturer’s protocol. Stained cells were analyzed as described above.

### Intravascular and tissue leukocyte discrimination

To characterize and differentiate lymphocytes residing within the affected tissue from those in vasculature, we performed intravascular staining of leukocytes [29]. In brief, Abs given via intravenous (i.v.) injection were diluted in sterile 1× DPBS and kept on ice protected from light. For the procedure, 3 μg of anti–CD8 α -PE (clone 53–6.7 from eBioscience) and anti–CD4-FITC (clone RM4-4 from eBioscience) in 300 μl of DPBS per mouse were used for intravenous injection. At 3 min after injection, the animals were sacrificed, and brain tissues were collected in RPMI1640 with 5% FBS. Brain mononuclear cells were prepared according to the above described protocol. Cells were washed to remove any excess of antibody. The cells were then stained *ex-vivo* with anti-CD8β-AF647 (clone YTS156.7.7 from eBioscience) and anti-CD4-AF700 (clone RM4-5 from eBioscience) for 30 min on ice. The cells were washed twice with FACS buffer and fixed using 2% paraformaldehyde for 30 min. Finally, the pellet was resuspended in 300 μl of FACS buffer for acquisition. Samples were acquired by flow cytometry and data were analyzed using FlowJo software (FlowJo, Ashland, OR).

### Statistical analysis

For comparing groups, two-tailed unpaired Student’s T-test for samples was applied, *p* values ≤ 0.05 were considered significant.

## Results

### Elevated proliferation of T-lymphocytes in the infected brain following Treg depletion

**Fig 1:**
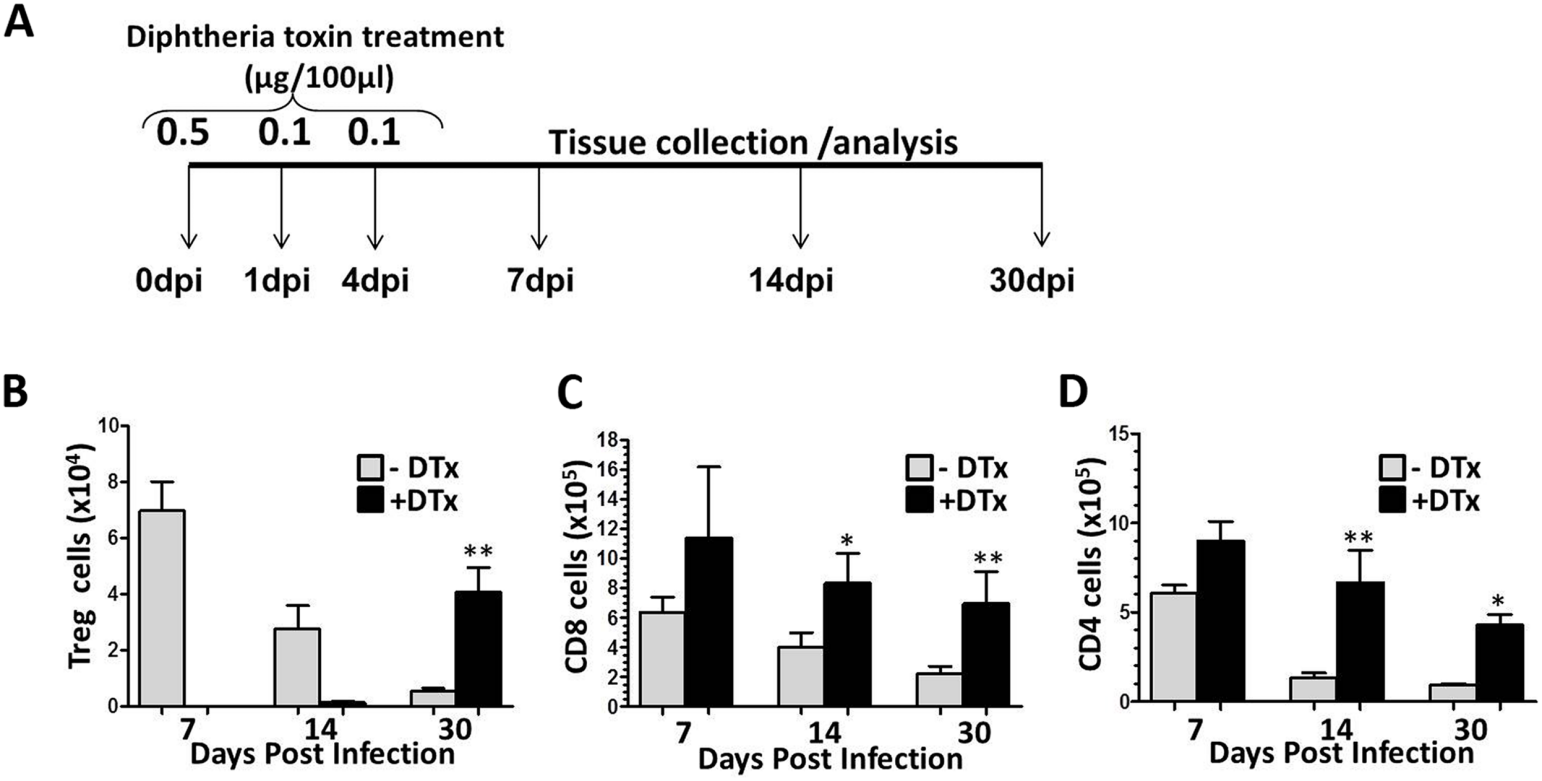
Elevated levels of T-lymphocytes within MCMV-infected brain following acute Treg depletion. (A) Schematic representation of diphtheria toxin injection given to Foxp3-DTR transgenic mice during acute phase of viral brain infection to deplete Treg cells. (B) Absolute numbers of Treg cells were calculated based on flow cytometric analysis from MCMV infected brain with and without DTx treatment at 7, 14, and 30 dpi. (C) Absolute numbers of CD8^+^ cells were determined within Dtx-treated and untreated, MCMV-infected brains at indicated the time points. (D) Absolute numbers of CD4^+^cells observed at the indicated time points. Pooled data are presented as mean ±SD of two experiments using six animals per treatment group/time point. *p < 0.01 and **p < 0.001 DTx- versus DTx+ MCMV-infected

Recent findings from our laboratory have demonstrated that following MCMV infection, regulatory T-cells are present within the CNS-infiltrating CD4^+^ T-lymphocyte population and accumulate within the brain from acute through chronic phases of infection [8]. In this study, using the well-established technique of *in-vivo* Foxp3^+^ cell ablation by administration of DTx to Foxp3-DTR animals (Fig 1A), we first assessed the presence of Tregs within the brains of both DTx-treated and untreated animals from acute through chronic phases of viral infection. As in our previous study, in DTx-untreated animals we observed an increased number of Treg cells within the brain, which decreased by d 30 p.i. However, in Treg-ablated animals, as the DTx was cleared from the host a small number of Tregs began to repopulate the brain by 14 dpi. A significant difference in Treg number was observed between the two groups as the Tregs repopulate the brain to even higher numbers than without DTx treatment by 30 dpi (Fig 1B). We also confirmed the effect of Treg ablation on the absolute number of brain T lymphocytes present within the brain. We observed an increased number of CD8^+^ and CD4^+^ T-cells within the brain of Treg-deficient, MCMV-infected mice when compared to infected, Treg-sufficient animals. There was no difference in the number lymphocytes at 7 dpi, while significant differences between the two groups were noted at the later time points (Fig 1C and 1D). These results were similar to those obtained in our previous studies.

**Fig 2:**
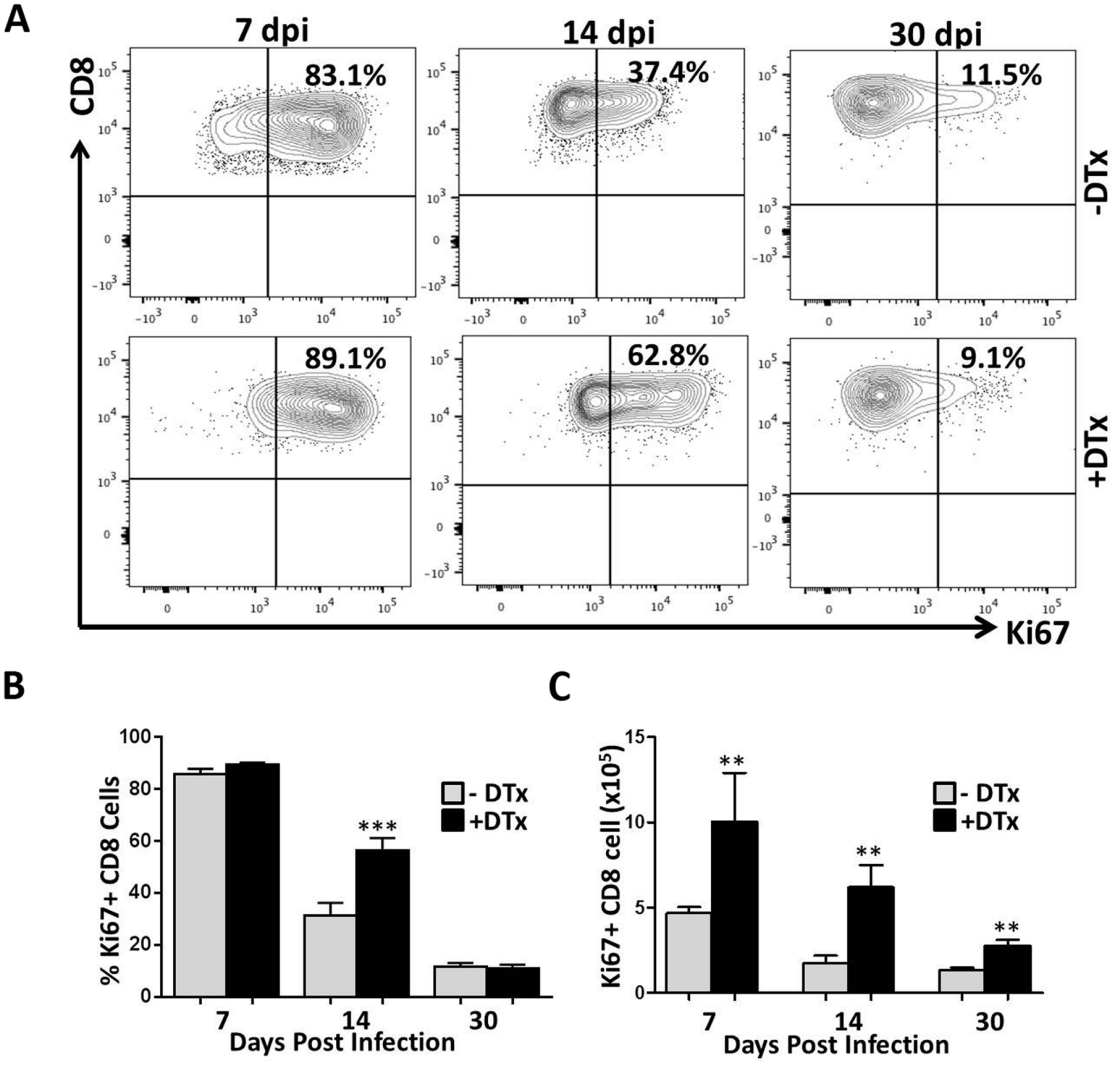
Enhanced proliferation of CD8^+^ T-lymphocytes in MCMV-infected brains following Treg depletion. (**A**) Single cell suspensions of brain tissue obtained from infected Foxp3-DTR transgenic mice (2–4 animals per time point) were collected and stained for flow cytometry with PE-Cy5-conjugated Abs specific for CD45, PE-Cy7-labeled for CD8, and Ki67 FITC–conjugated Abs. An isotype Ab for Ki67 FITC was used as gating control. Contour plots showing the proliferation frequency of CD8^+^ T-cells from infected, untreated (-DTx), and DTx-treated (+DTx) animals at the indicated time points are representative of 2 independent experiments. (B) Pooled data show percentage proliferation of CD8^+^ T-cells (mean ±SD) in infected brains with and without treatment at the indicated time points. ***p < 0.0001 DTx- versus DTx+ MCMV-infected at 14 dpi. (C) The number of proliferating CD8^+^ T-cells in infected brains of animals with and without DTx treatment at the indicated time points is shown. **p < 0.001 DTx- versus DTx+ MCMV-infected animals

**Fig 3:**
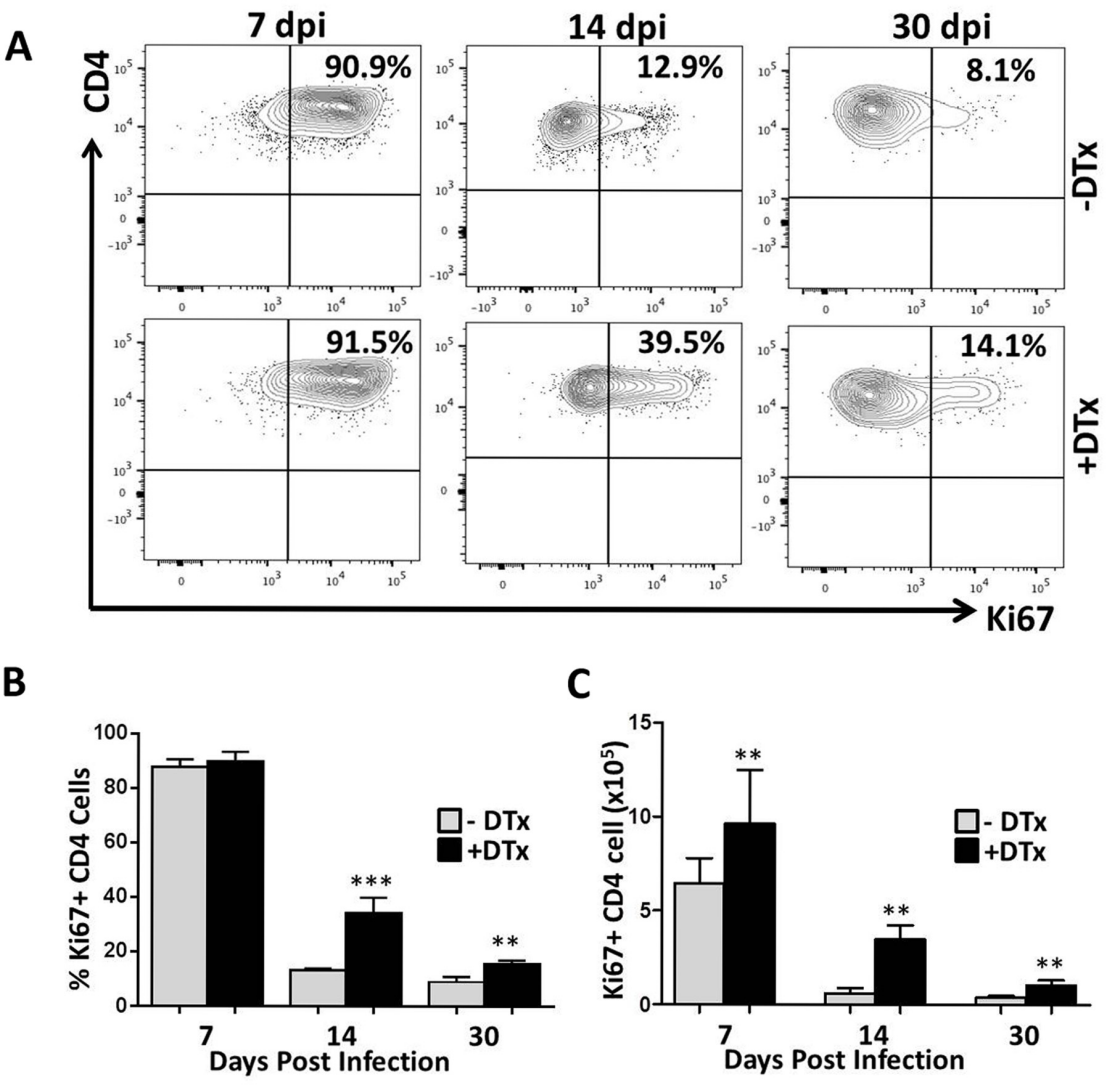
Increased proliferation of CD4^+^ T-lymphocytes in the infected brains following Treg depletion. (A) Brain tissue from MCMV-infected, Foxp3-DTR transgenic mice was collected at 7, 14, and 30 dpi. Brain-infiltrating leukocytes were isolated and stained for flow cytometry with PE-Cy5-conjugated Abs specific for CD45, PE-Cy7-labeled for CD4-e-F 450, and Ki67 FITC–conjugated Abs. Isotype Abs for Ki67 FITC were used as gating control. Contour plots showing the proliferation frequency of CD4^+^ T-cells from infected, untreated (-DTx), and DTx-treated (+DTx) animals at the indicated time points are representative of 2 independent experiments. (B) Pooled data show percentage proliferation of CD4^+^ T-cells (mean ±SD) in infected brains with and without treatment at the indicated time points. ***p < 0.0001 DTx- versus DTx+ MCMV-infected at 14 dpi. **p < 0.001 DTx- versus DTx+ MCMV-infected at 30 dpi. (C) The number of proliferating CD4^+^ T-cells in infected brains of animals with and without DTx treatment at the indicated time points is shown. **p < 0.001 DTx- versus DTx+ MCMV-infected animals

Given the differences in T-lymphocyte number within MCMV-infected brains following Treg depletion, it was important to first evaluate if loss of Tregs altered the proliferation of infiltrating T-lymphocytes in response to MCMV brain infection. We examined single cell suspensions of infected brain tissue with and without the DTx treatment using multi-color flow cytometry. We observed increased proliferation (i.e., Ki67 staining) of CD8^+^ and CD4^+^ T-cells within brains of Treg-deficient, MCMV-infected mice when compared to Treg-sufficient animals (Figs 2A and 3A, respectively). Analysis of pooled data showed differences in proliferation were significant for both CD8^+^ and CD4^+^ T-cell populations at 14 dpi (P<0.0001 for both CD8^+^ and CD4^+^ T-cells), whereas Treg ablation generated no significant differences in either CD8^+^ and CD4^+^ T lymphocyte proliferation at 7 dpi. At 30 dpi, differences in CD8^+^ T-cell proliferation were not detected, but CD4^+^ T-cell proliferation was significantly elevated (P<0.001) within brains of DTx-treated animals (Figs 2B and 3B, respectively). Evaluation of the absolute numbers of proliferating CD8^+^ and CD4^+^ T-cells revealed robust differences between the two groups at each time point tested (Figs 2C and 3C, respectively).

To examine whether DTx treatment alone had an effect on infection-induced brain inflammation [30], we treated wild-type C57B/6 mice with the same dose of DTx and examined the proliferation of lymphocytes in these wild-type animals with and without DTx treatment. In these studies, we observed no effect of DTx treatment on proliferation of either CD8^+^ or CD4^+^ T lymphocytes in wild-type animals (S1A, S1B and S1C Fig). Additionally, the development of CD103^+^ cells in response to viral infection was equivalent in wild-type animals with or without DTx treatment (S1D Fig).

We also found elevated levels of CD8^+^ T-cell proliferation within the draining cervical lymph nodes of DTx-treated mice at 7 dpi and 14 dpi (i.e., 38.9% vs. 13.3%, and 26.0% vs. 8.31% with and without DTx, respectively), (S2 Fig). Similar observations were made for CD4^+^ T-cells within the lymph nodes at 7 dpi and 14 dpi (i.e., 66.7% vs. 16.3%, and 41.4% vs. 11.4% with and without DTx, respectively), (S3 Fig). Interestingly, CD8^+^ T-cell proliferation within the draining lymph nodes decreased by 30 dpi regardless of DTx treatment (5.46% vs. 8.39%, with and without DTx, respectively); whereas elevated CD4^+^ T-cell proliferation in treated animals was still observed at 30 dpi (14.8% vs. 7.18% with and without DTx, respectively).

### Discrimination between brain tissue localized and vascular lymphocyte proliferation

**Fig 4:**
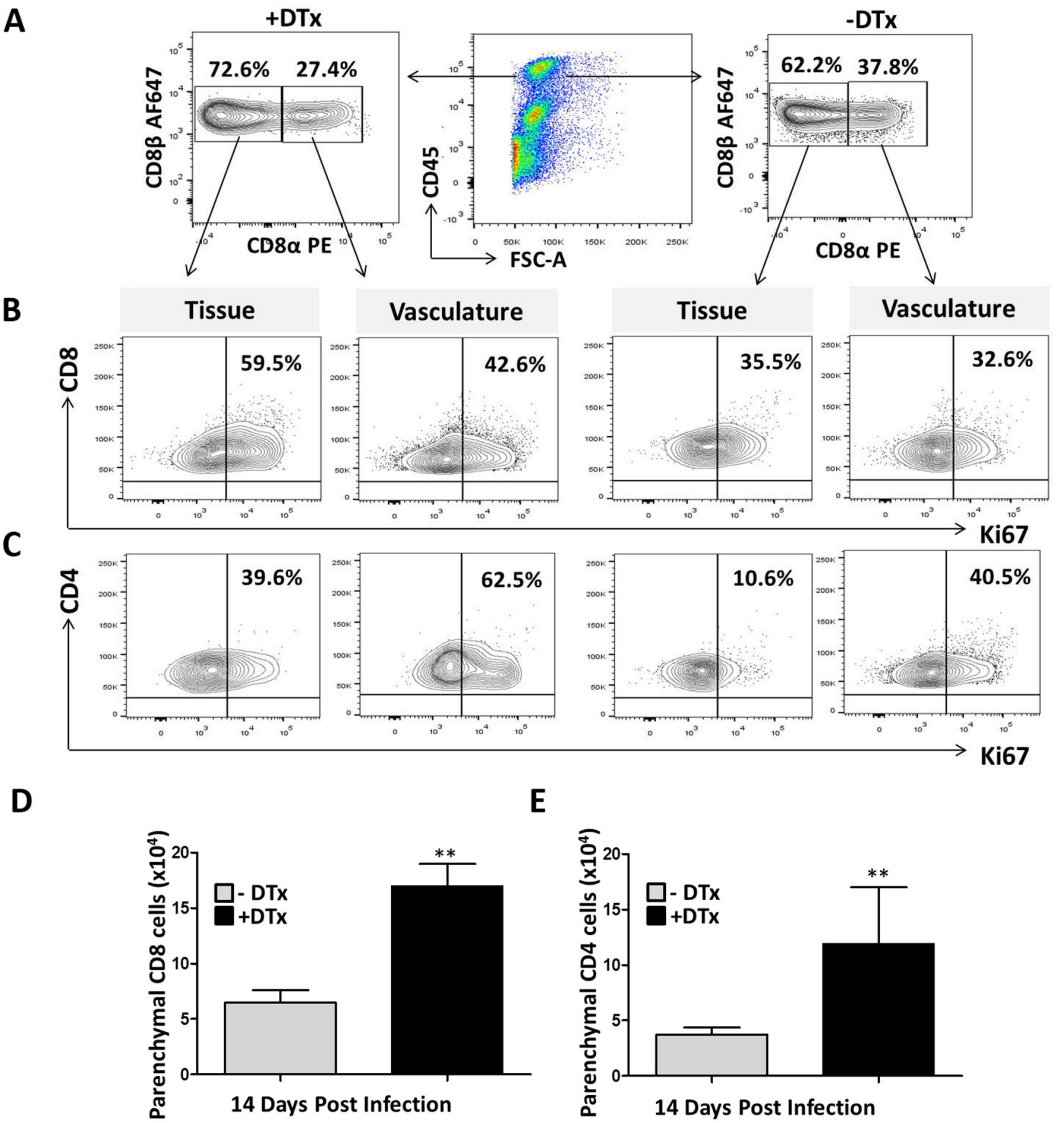
Discrimination between brain parenchyma-localized and vasculature-localized lymphocyte proliferation. Foxp3-DTR, MCMV-infected, DTx-treated and untreated mice at 14 dpi were injected intravenous with anti-CD8α-PE and anti-CD4-FITC mAb. Lymphocytes were isolated and stained *ex-vivo* for the anti-CD8 β-AF647 and anti-CD4-AF700 using different clones, as described in the methods. Plots are representative of two experiments using three animals per groups. (A) Contour plots show CD8^+^ T-cells in the vasculature that stained both for anti-CD8α-PE and anti-CD8β-AF647; while tissue lymphocytes were stained by anti-CD8β-AF647 alone in both DTx-treated and untreated groups. (B) Contour plots show proliferation of CD8^+^ T-cells both within the tissue and in the vasculature. **C.** Contour plots represent proliferation of CD4^+^ T-cells both in tissue and vasculature. (D) The number of parenchyma-localized CD8^+^ T-cells within MCMV-infected brains of animals with and without DTx treatment is shown. (E) The number of parenchymal CD4^+^ T-cells with and without DTx treatment is shown. **p < 0.001 DTx- versus DTx+ animals

Discrimination between brain tissue-localized lymphocytes and those within the vasculature is important not only to evaluate immune cells within the brain parenchyma, but also to understand the role of these cells within the vessels. Because we first observed the kinetics of lymphocyte proliferation in whole brain tissue obtained from DTx-treated and untreated animals, we went on to characterized lymphocyte proliferation status within the parenchyma. For this we adapted a method of intravascular staining previously developed by other investigators [29, 31]. Using this approach, we employed, MCMV-infected, DTx treated and untreated FoxP3-DTR mice animals at 14 dpi when infiltration of leukocytes into the brain is negligible. The vascular population of CD8^+^ T-cells were identified by dual staining with anti–CD8a-PE (clone 53–6.7), administered by intravenous injection, and anti-CD8a-AF647 (clone YTS156.7.7) stained *ex-vivo*. Similarly, CD4^+^ T -cells were distinguished by the co-stained population using anti-CD4-AF700 (clone RM4-5) and anti–CD4-FITC (clone RM4-4). Leukocytes residing within the brain were identified by *ex-vivo* single staining (Fig 4A). Next, the distinguished populations of vascular and tissue lymphocytes were further characterized for their proliferation by Ki67 staining. In these studies, we observed that 35.5% of CD8^+^ T-cells and 10.6% of CD4^+^ T- cells in the brain tissue; and 32.6% of CD8^+^ T cells and 40.5% of CD4^+^ T- cells in the vasculature were positive for Ki67 in DTx-untreated animals. The lymphocytes in DTX-treated animals showed higher levels of proliferation with 59.5% of CD8^+^ T- cells and 39.6% of CD4^+^ T-cells in the brain tissue, and 42.6% of CD8^+^ T cells and 62.5% of CD4^+^ T- cells in the vasculature expressing Ki67 (Fig 4B and 4C). Thus, these data indicate that both CD8^+^ and CD4^+^ T lymphocytes proliferated within the brain parenchyma upon viral infection, with CD8^+^ T-cells showing higher levels. We also examined the number of parenchyma-localized CD8^+^ and CD4^+^ T lymphocyte in Treg-deficient and Treg-sufficient animals. Analysis of pooled data (n = 6 animals per group, two different experiments) revealed elevated levels of both types of T-cells in DTx treated animals when compared to untreated mice (Fig 4D and 4E).

### Tregs restrain activation and cytokine production by T lymphocytes within MCMV-infected brains

**Fig 5:**
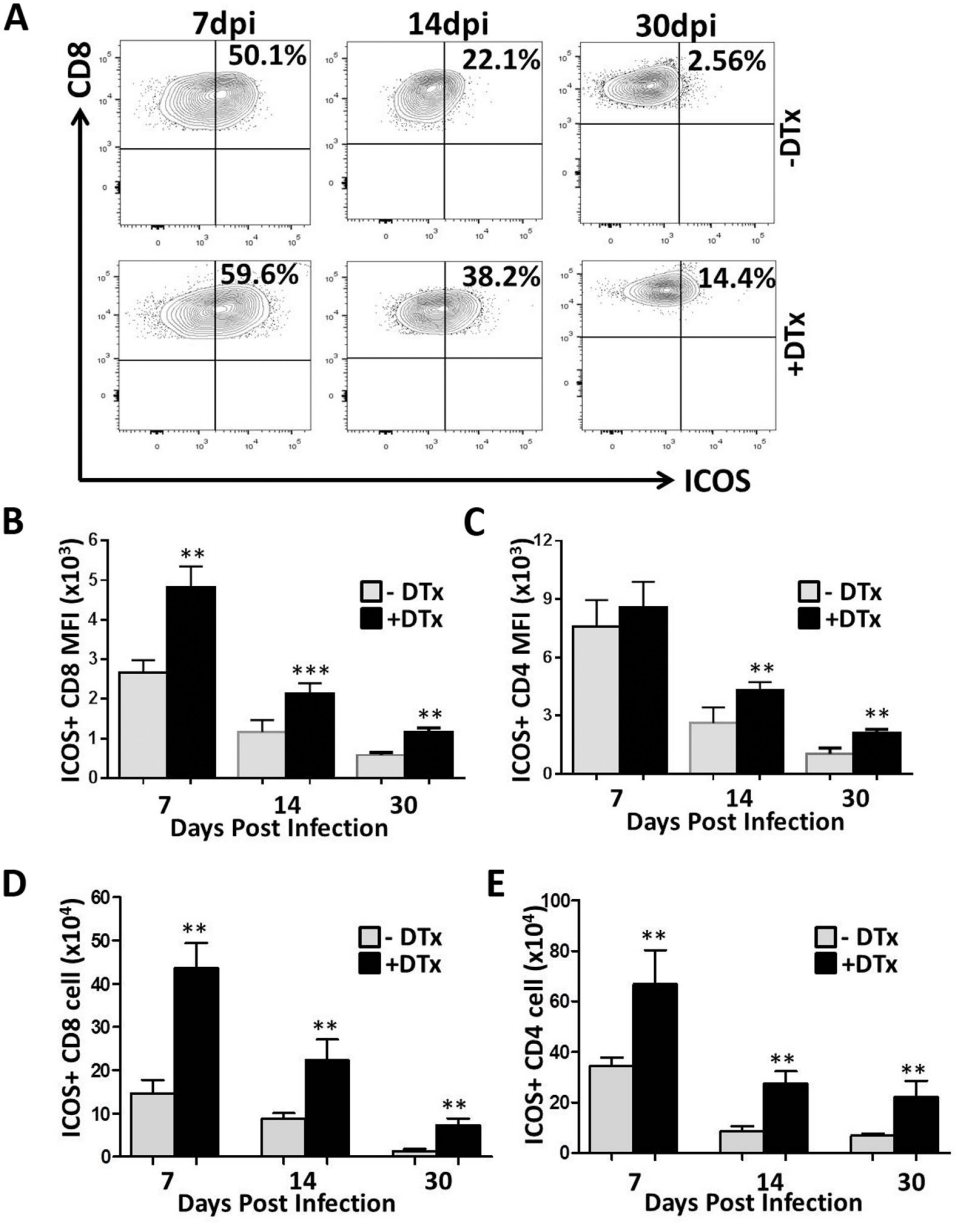
Tregs limit T-lymphocyte activation within MCMV-infected brains. Activation of CD8^+^ and CD4^+^ T-cells was evaluated based on flow cytometric analysis using PE-Cy5-labeled inducible co-stimulatory molecule (ICOS) Abs. (A) Representative contour plots show the percentage of CD8^+^ T-cells expressing the activation marker ICOS from infected, untreated, as well as DTx-treated animals at the indicated time points. (B) Data presented show the mean fluorescent intensity (MFI) of ICOS expression on CD8^+^ T-cells (mean ±SD) between groups. **p <0.001 DTx- versus DTx+ MCMV-infected at 7 & 30 dpi and ***p<0.0002 at 14 dpi. (C) Pooled data presents the mean fluorescent intensity (MFI) of ICOS expression on CD4^+^ T-cells (mean ±SD) between groups. **p <0.001 DTx- versus DTx+ MCMV-infected at 14 & 30 dpi. (D) The number of ICOS^+^CD8^+^ T-cells in infected brains with and without DTx treatment at the indicated time points is shown. (E) The number of ICOS^+^CD4^+^ T-cells in infected brains with and without DTx treatment at the indicated time points is shown. **p < 0.001 DTx- versus DTx+ animals

Having determined differences in lymphocyte proliferation in the absence of Tregs, we went on to determine their role in limiting T-cell activation. Here, we measured the expression level of Inducible costimulatory molecule (ICOS) on lymphocytes within the brain, discriminating their activation status during different phases of infection. ICOS, a member of the CD28/CTLA4 family, has been reported to be essential for T-cell activation and function in inflammatory diseases. It has also been reported that ICOS knockout mice are less efficient in mounting T-cell-dependent immune responses in vivo [32, 33]. Our study showed that both CD8^+^ and CD4^+^ T-cells were highly activated within the brains of Treg-deficient animals, when compared to Treg-sufficient mice, with CD8^+^ T-cells showing significant differences at every time point examined (i.e., P<0.001 at 7 dpi, P<0.0002 at 14dpi, and P<0.001 at 30 dpi), (Fig 5A and 5B). The difference in activation for CD4^+^ T-cells was found to be significant at 14 and 30, but not 7 dpi (P<0.001, Fig 5C). We also evaluated the absolute number of T-cells expressing ICOS between the groups. We observed an increased number of activated lymphocytes in the brains of Treg-ablated animals when compared to Treg-sufficient mice. Differences were found to be significant for both lymphocyte subsets at each time points (P < 0.001, Fig 5D and 5E).

**Fig 6:**
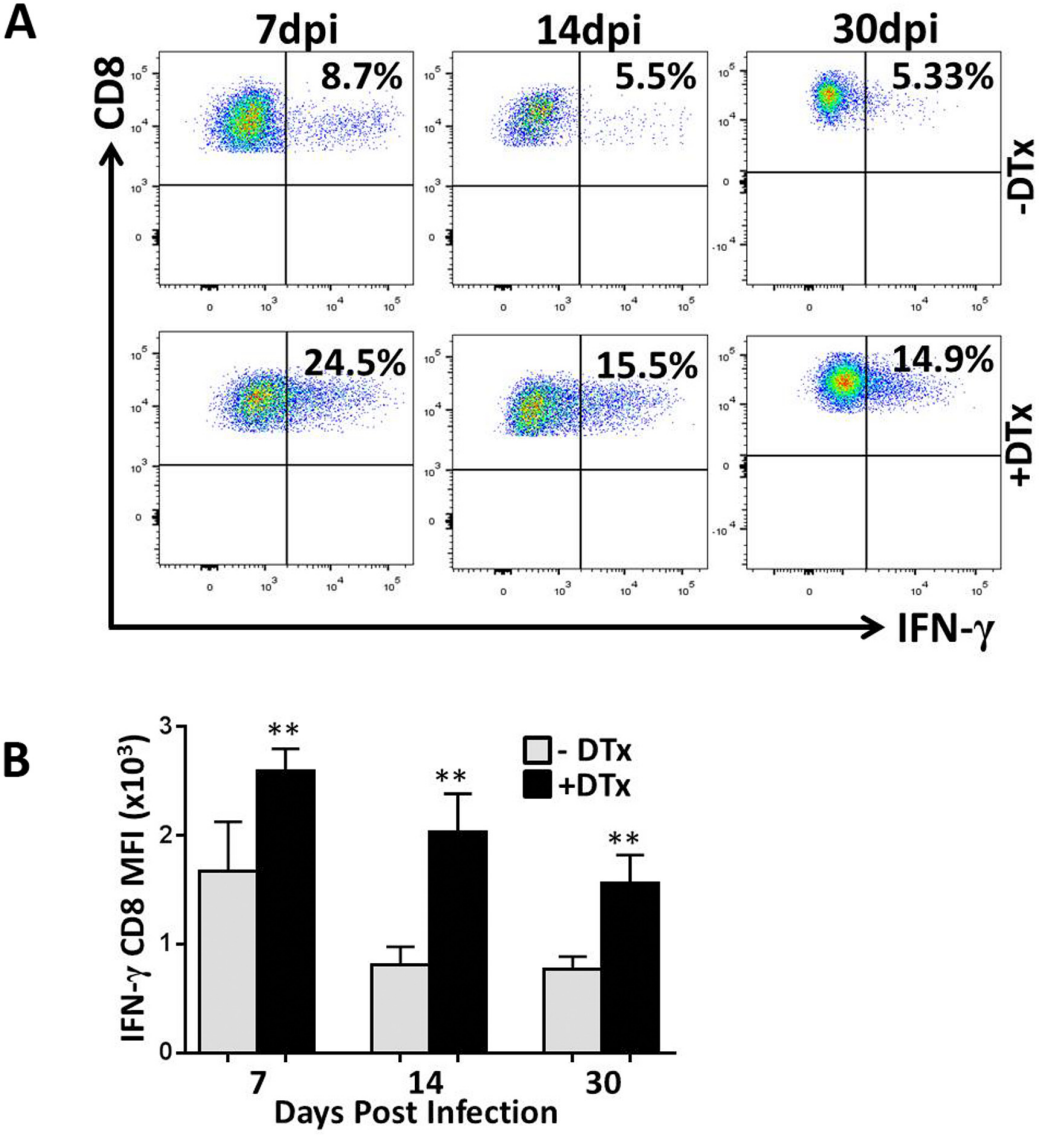
Tregs limit IFN-γ production by T lymphocytes within MCMV-infected brains. Single cell suspension of brain tissue obtained from MCMV-infected DTx-treated and untreated mice (2–4 animals per time point) were banded on a 70% percoll cushion. Brain leukocytes at the 30–70% percoll interface were collected. For intracellular IFN-γ staining, brain leukocytes (2 x 10^6^ cells/ml) were pulsed with either anti-CD3/CD28 antibodies or with a MCMV-specific, MHC class 1-restricted M45 peptide (6 h at 37°C) and treated with Brefeldin A. After incubation, cells were washed in FACS buffer and stained for the surface molecules CD45, CD8, and for intracellular IFN-γ using a Cytofix/Cytoperm kit (BD Pharmingen), before flow cytometry. (**A**) Representative plots shows the ratio of CD8^+^ T lymphocytes producing IFN-γ among the DTx-treated (+DTx) and untreated (-DTx) groups at 7,14, & 30 dpi in response to peptide treatment. (B) Pooled data show the (mean ±SD) of CD8^+^ T-cells producing IFN-γ at the indicated time points from 2 independent experiments. **p < 0.001 DTx- versus DTx+ MCMV-infected at 7, 14, & 30 dpi.

**Fig 7:**
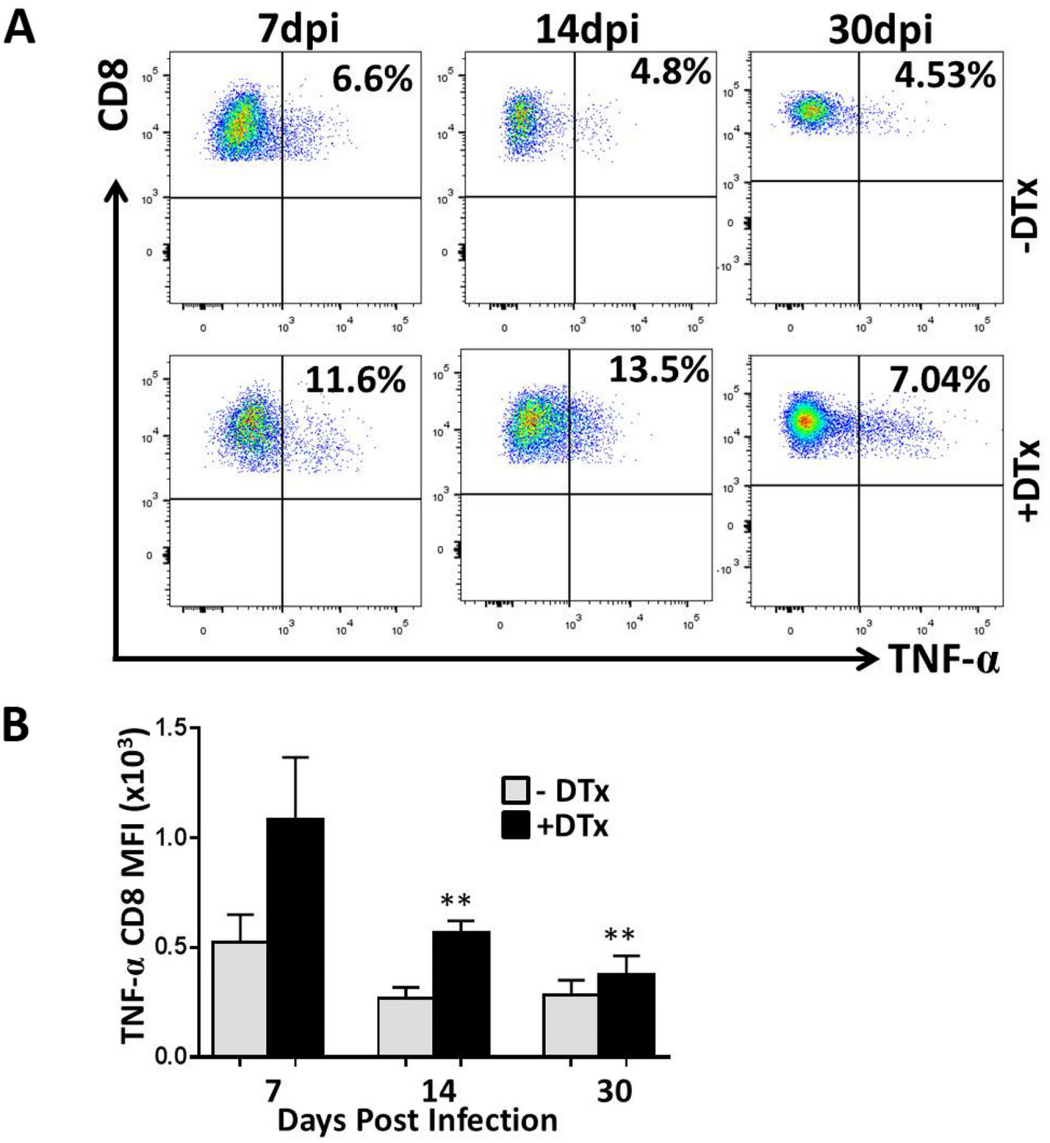
Tregs attenuate TNF-α production by T lymphocytes within MCMV-infected brains. For intracellular staining of TNF-α, brain leukocytes (2 x 10^6^ cells/ml) were pulsed with either anti-CD3/CD28 antibodies or with an MCMV-specific, MHC class 1-restricted M45 peptide (6 h at 37°C) and treated with Brefeldin A. After incubation, cells were washed in FACS buffer and stained for the surface molecules CD45, CD8, and for intracellular staining of TNF-α using a Cytofix/Cytoperm kit (BD Pharmingen), before flow cytometry. (A) Representative plots show the ratio of CD8^+^ T lymphocytes producing TNF-α among the DTx-treated (+DTx) and untreated (-DTx) groups at 7,14, & 30 dpi in response to peptide treatment. (B) Pooled data show the (mean ±SD) of CD8^+^ T-cells producing TNF-α at the indicated time points from 2 independent experiments. **p < 0.001 DTx- versus DTx+ MCMV-infected at 14 dpi and 30 dpi.

**Fig 8:**
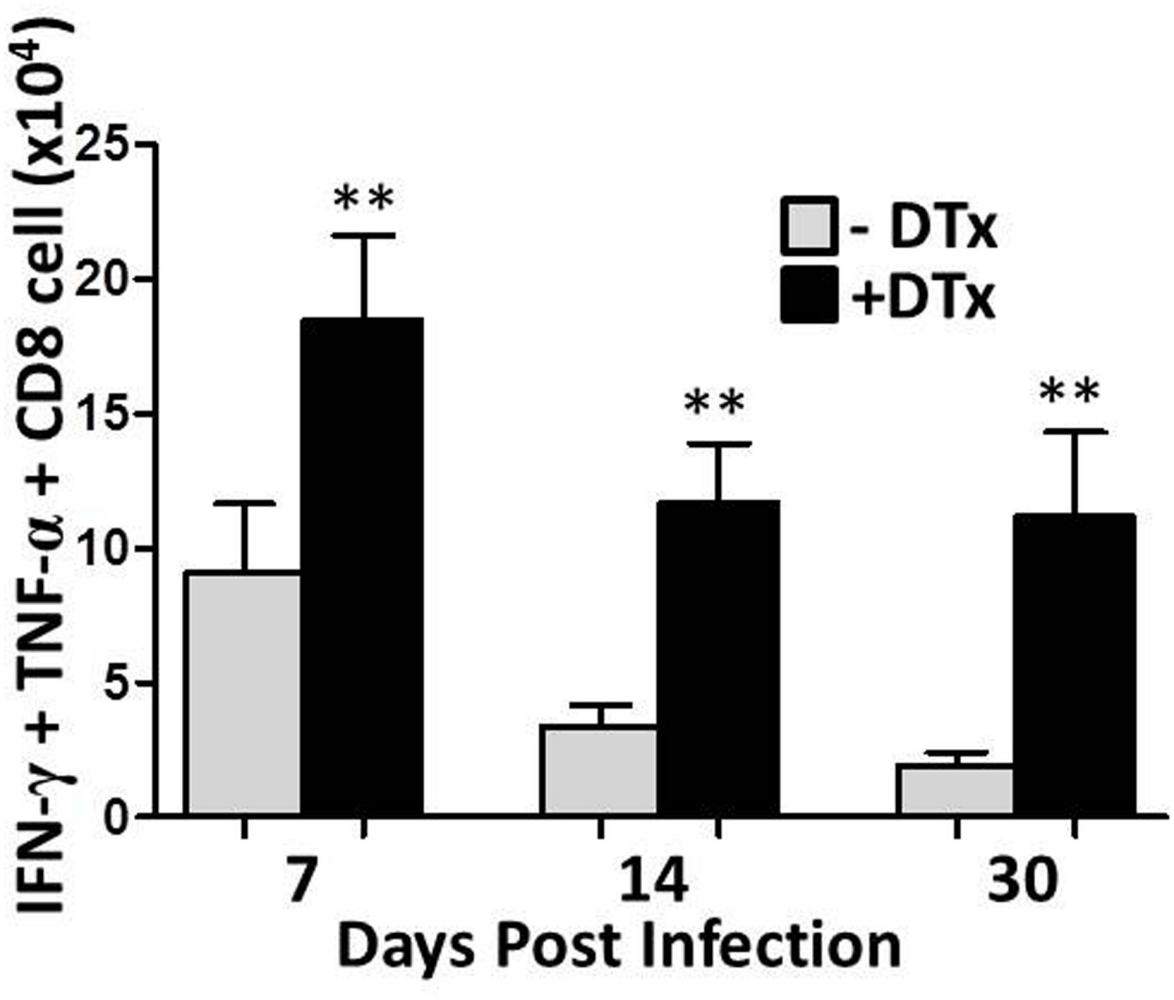
Treg depletion results in an increased number of CD8^+^T-cells displaying dual cytokine production. Pooled data show absolute numbers of CD8^+^ T-cells producing both IFN-γ and TNF-α among the DTx-treated (+DTx) and untreated (-DTx) groups at 7,14,and 30 dpi in response to peptide stimulation. Data are from 2 independent experiments using 2–4 animals per treatment group/time points. **p < 0.001 DTx- versus DTx+ MCMV-infected at 7, 14, & 30dpi

Studies from our laboratory have previously demonstrated that MCMV brain infection results in long-term persistence of antigen-specific CD8^+^ T-cells which may contribute to chronic microglial cell activation [4]. To further investigate the role of Tregs and to assess their significance, we then studied the functional ability of CD8^+^ T-cells to respond to re-stimulation. This was done by performing intracellular cytokine staining to evaluate cytokine production by cells specific for the MCMV peptide M45, which has previously been identified as MCMV-specific major histocompatibility complex (MHC) class I restricted epitope [27, 28]. Brain mononuclear cells derived from infected brain at different time points were incubated with either CD3/CD28 antibodies for control, or MCMV M45 peptide for 5 h, then subjected to intracellular staining for IFN-γ and TNF-α after immunostaining for appropriate surface markers. In these studies, the frequency of CD8^+^ T-cells producing IFN-γ and TNF-α was found to be increased in Treg-ablated, MCMV-infected mice when compared to Treg sufficient animals (Figs 6A and 7A). These differences reached statistical significance at each time point for IFN-γ (P<0.001, Fig 6B), and at 14 and 30 dpi for TNF-α (P<0.001, Fig 7B). Thus, these data show elevated proinflammatory cytokine production following Treg depletion. Additionally, we also evaluated the number of CD8^+^ T-cells that were polyfunctional in terms of their ability to produce both IFN-γ and TNF-α. We found that in Treg-deficient mice, there were more CD8^+^ T-cells displaying dual cytokine production when compared to Treg-sufficient animals (Fig 8). The differences were found to be significant at each time point, indicating a more proinflammatory environment in Treg-deficient animals.

### Treg proliferation and activation during MCMV infection

**Fig 9:**
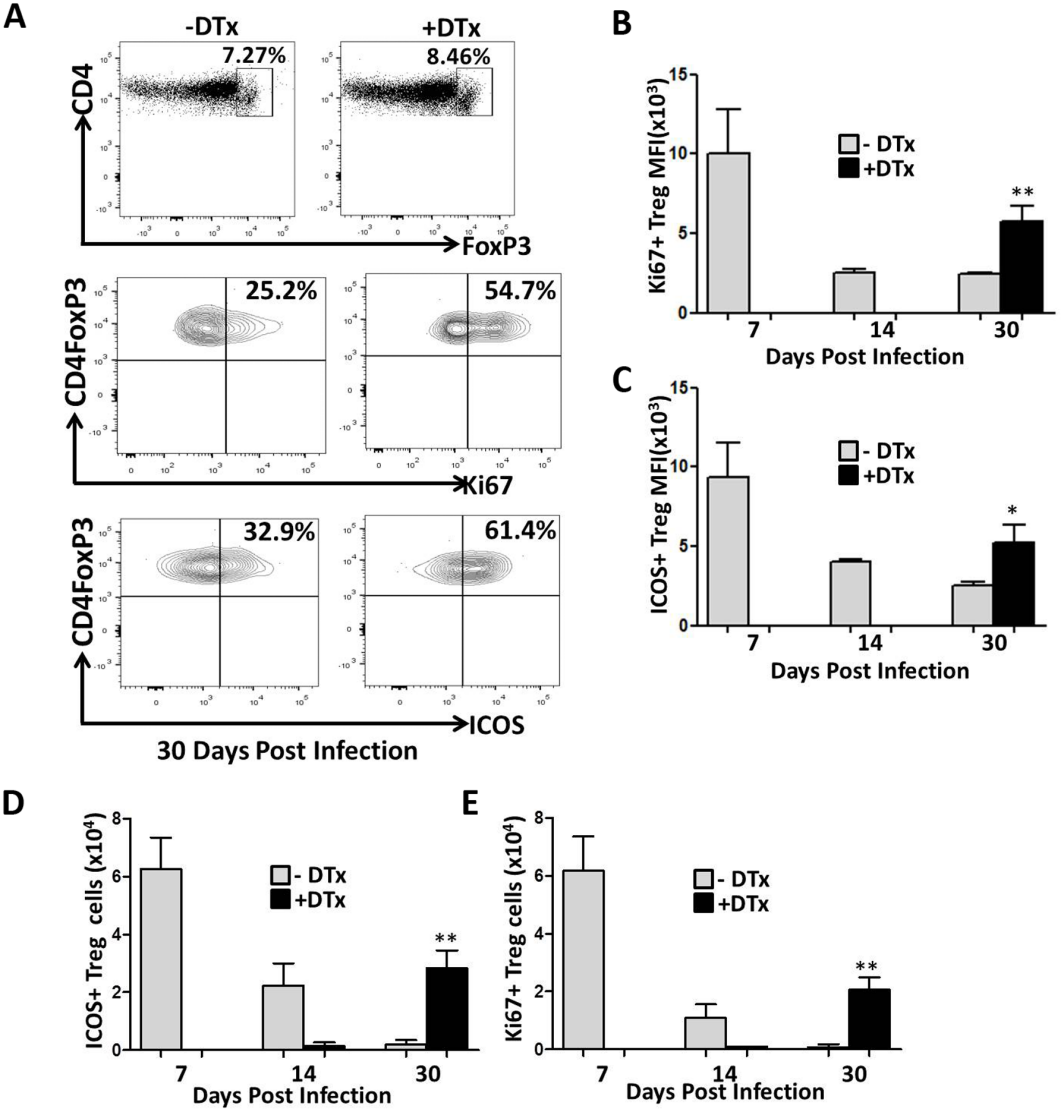
Treg proliferation and activation during MCMV infection. (A) Brain tissue from MCMV-infected, DTx-treated and untreated Foxp3-DTR transgenic mice were collected at 30 dpi. Brain-infiltrating leukocytes were isolated and stained for flow cytometry. CD4^+^ T-cells were gated on from the CD45^+^CD11b^low^ leukocyte population for analysis of Foxp3 expression. Isotype control Abs for Foxp3 was used as a gating control. Plots show Ki67-positive and ICOS-expressing Tregs, and are representative of two separate experiments using four animals/time point. (B) Data show the MFI of Ki67-expressing Tregs from the DTx-treated and untreated groups at the indicated time points from 2 independent experiments. **p = 0.0047 DTx- versus DTx+ MCMV-infected at 30 dpi. (C) Pooled data present the MFI of ICOS expression on Tregs between groups at the indicated time points. *p = 0.0165 DTx- versus DTx+ MCMV-infected at 30 dpi. (D) The number of ICOS^+^ Treg cells in infected brains with and without DTx treatment at the indicated time points is shown. **E.** The number of Ki67^+^Treg cells in infected brains with and without DTx treatment at 7, 14, & 30 dpi is shown. **p < 0.001 DTx- versus DTx+

Previous findings have demonstrated that Tregs infiltrate the CNS between 4 and 7 d following MCMV brain infection, indicating that accumulation of these cells may play a regulatory role in controlling immunopathogenesis. We next investigated the expansion and activation of the Treg cells themselves in response to MCMV brain infection. Data obtained show that Tregs within the brains of infected animals without DTx-treatment expanded gradually from the acute phase through chronic phase of infection. However, Treg cell proliferation was found to be more rapid during the acute phase infection when the percentage of Tregs was low. Proliferation of Treg cells as indicated by Ki67 staining was found to decrease by 30 dpi in DTx-untreated animals. In contrast, when Tregs were ablated during acute infection, their numbers rebounded by 14–20 dpi, with significantly increased proliferation at d 30 p.i. (P = 0.0047), (Fig 9A and 9B). Similarly, marked differences in the expression level of ICOS were observed between DTx-treated and untreated groups, indicating that Tregs which repopulate the brain were highly activated (Fig 9A and 9C). In contrast, in untreated groups ICOS expression on Tregs progressively decreased by 30 dpi (P = 0.0165). In addition, we evaluated the number of Treg cells that were activated and proliferative. We observed that in DTx-untreated animals, ICOS expressing Treg cells were present within the brain during the acute phase of infection, the number of which decreased by 30 dpi. Similar observations were made in proliferating Treg cells. In contrast, the number of activating and proliferating Treg cells which rebounded by 30 dpi was significant higher in DTx-treated animals (Fig 9D and 9E).

### Effect of Treg ablation on effector to memory cell transition and T_RM_ cell accumulation within the infected brain

**Fig 10:**
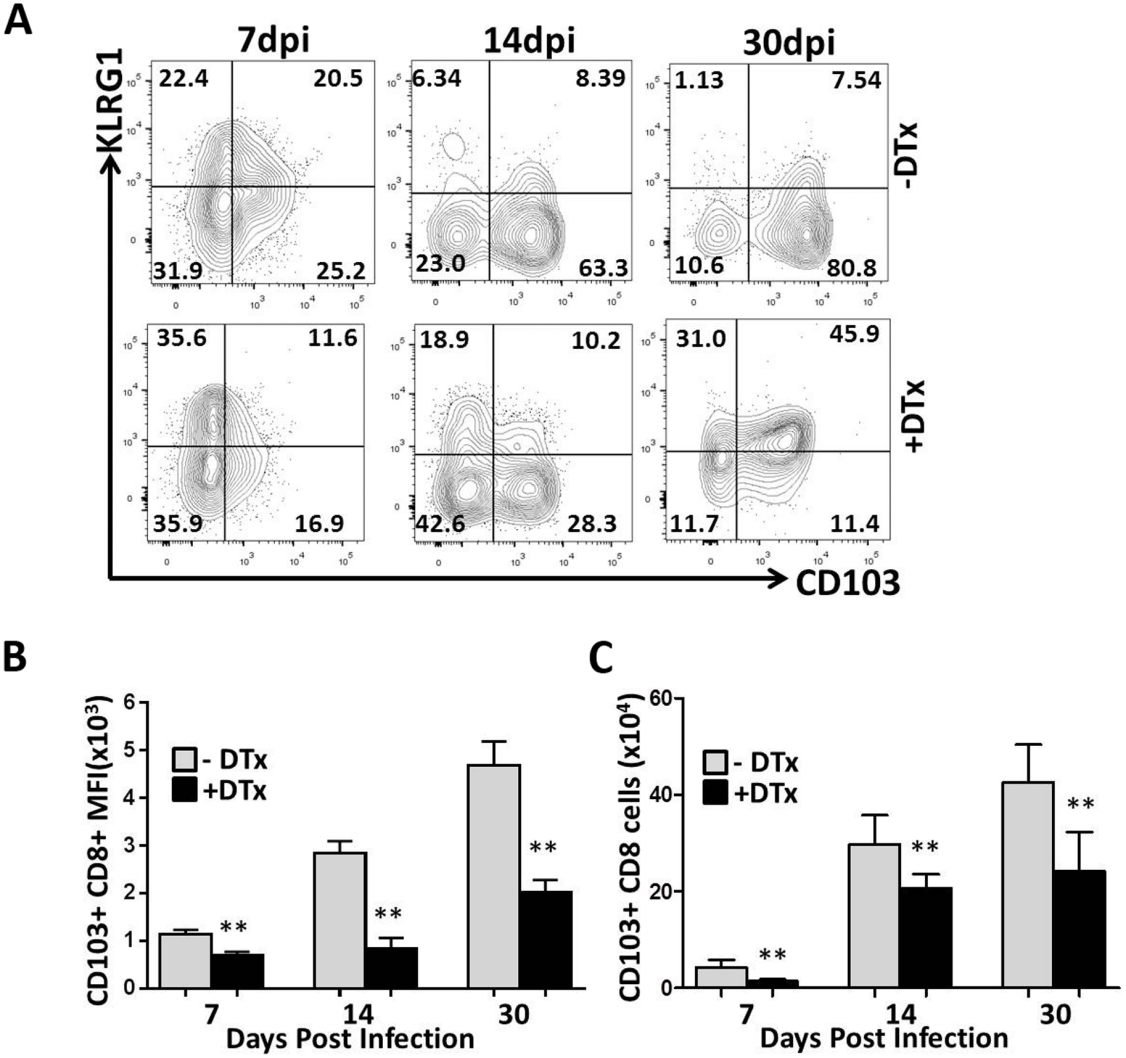
Tregs modulate retention of T_RM_ cells within the brain following MCMV infection. Mononuclear cells were harvested from Treg-sufficient and deficient mice, as previously described at 7, 14, & 30 dpi. CNS-derived lymphocytes were gated on CD8^+^ T-cells and analyzed for KLRG1 and CD103 (a marker for tissue resident-memory T-cells in the CNS). (A) Representative contour plots show expression levels of KLRG1 and CD103 gated on CD8^+^ T-cells at the indicated time points. (B) Pooled data show the MFI of CD8^+^ T-cells expressing CD103 at the indicated time points. Data are derived from 2 independent experiments using 2–4 animals per treatment group/time point. **p < 0.001 DTx- versus DTx+ MCMV-infected at all time points tested. (C) The number of CD103^+^CD8^+^ T-cells in infected brains with and without DTx treatment at 7, 14, &30 dpi is shown. **p < 0.001 DTx- versus DTx+

During viral infection naive CD8^+^T-cells rapidly proliferate and differentiate to form both short-lived effector cells and a small subset of long-lived memory cells. In addition, it is also well established that circulating effector-memory T-cells do not normally access immune privileged tissues like the brain during routine immune surveillance. Thus, generation of long-lived memory cells which permanently reside within the tissue may be critical for defense of immune-privileged sites. Memory T-cells which reside permanently within a tissue and do not circulate are termed tissue resident -memory (T_RM_). In the brain, these cells have been characterized by marked elevated expression of CD103, a marker of T_RM_ within the CNS [7, 34, 35]. We next hypothesized that T_RM_ cells may be of particular importance within the brain, so we characterized the role of Tregs in transition of short-lived effector cells (SLECs), formed during acute phase of infection, to a pool of long lived memory cells (referred to as memory precursor cells, MPECs). As reported by other investigators, heterogeneity in the expression of killer cell-like lectin receptor G1 (KLRG1) and CD127 distinguishes between SLECs and MPECs. Long-lived circulating memory precursor cells are derived from KLRG1^-^CD127^hi^ cells, while the KLRG1 high population represents short-lived effector cells [13, 14, 36]. Examination of these distinct surface markers by flow cytometry revealed striking differences within the brains of our MCMV-infected animals. During acute phase, in both Treg-depleted and Treg-sufficient animals KLRG1^+^ CD8^+^ T-cells were present at high levels (Fig 10A). In contrast, negligible expression of KLRG1 but increased expression of CD127 was noted at 30 dpi in Treg-sufficient animals. We further characterized the phenotype of T_RM,_ and determined the expression of CD103 on CD8^+^ T-cells at various time points post-infection. We observed progressive upregulation of CD103 and downregulation of KLRG1 from7dpi to 30 dpi (Fig 10A). T_RM_ cells are known to be derived from the KLRG1^-^ precursors and this finding is in accordance with similar reports [14, 37–39]. When data generated using Treg-sufficient animals was compared to those obtained with Treg-deficient mice, we found significantly fewer CD103^+^CD8^+^ T-cells within the brain of DTx-treated animals. This difference was found to be statistically significant at each time points post-infection (Fig 10B). Additionally, in Treg-depleted animals, KLRG1 expression was still evident at 30 dpi; however, its expression had decreased in Treg-sufficient groups at this late time point (Fig 10A). Finally, a steady increase in the expression level of CD127 from effector phase through to the memory state was seen in in both groups (S4 Fig). We also determined the numbers of CD103^+^CD8^+^ T-cells in both Treg-sufficient and deficient animals. Again, more CD103^+^CD8^+^ T-cells were found in the brain of animals with Treg cells in contrast to those without Tregs and the differences were found to be significant at each time point (Fig 10C). Importantly, in draining lymph nodes there were few CD103^+^CD8^+^ T-cells, even though KLRG1 expression decreased by 30 dpi, expression of CD103 in cervical lymph nodes was not different with or without Treg depletion (S5 Fig).

### Decreased granzyme B production upon antigen re-stimulation by T_RM_ cells from Treg-depleted animals

**Fig 11:**
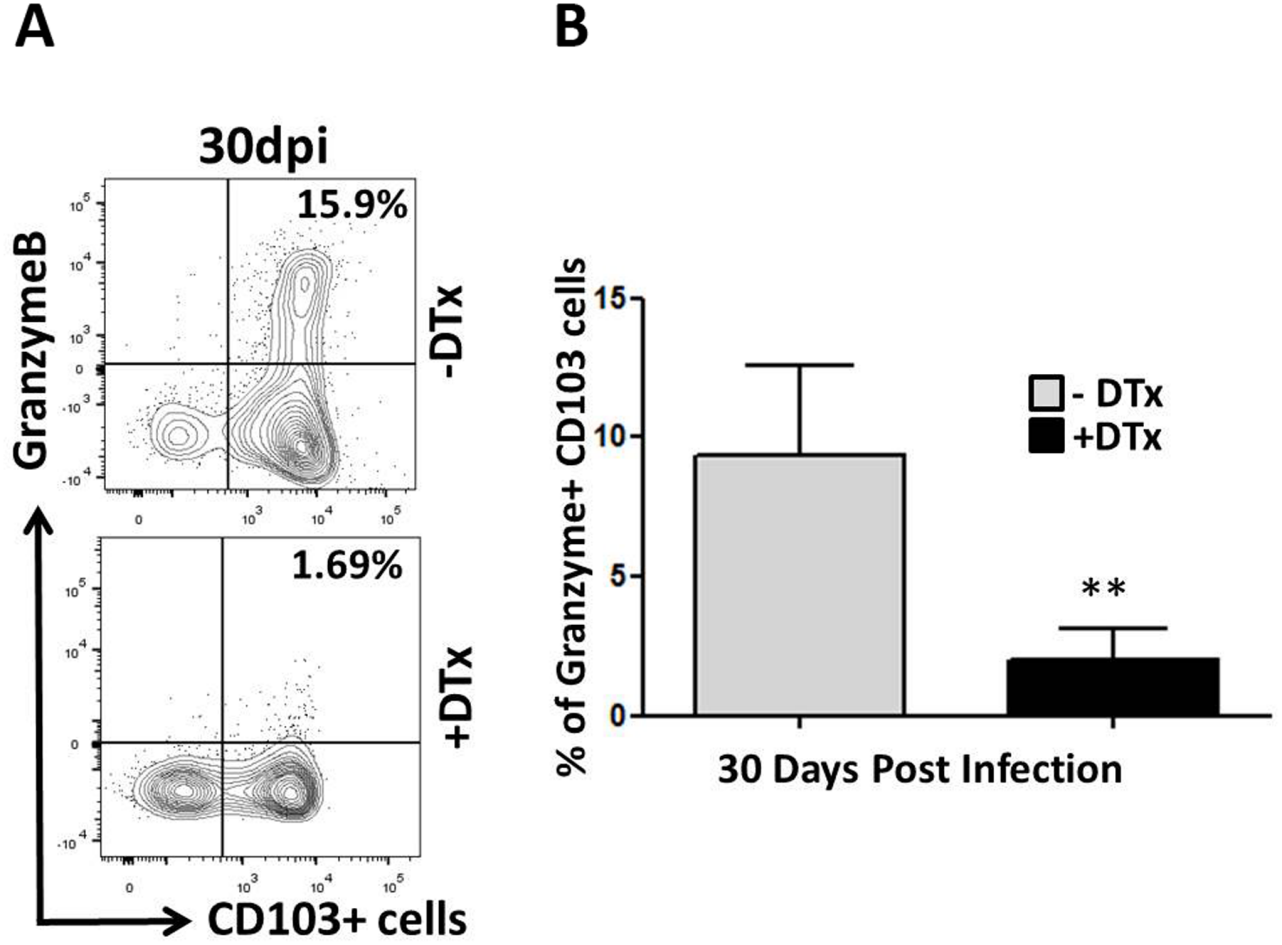
Decreased granzyme B production upon antigen re-stimulation by T_RM_ cells from Treg-depleted animals. Single cell suspensions of brain tissue obtained from MCMV-infected mice with and without Treg ablation were re-stimulated using the MCMV-specific peptide M45 (6 h at 37°C). Cells were stained for flow cytometry and granzyme B production was analyzed in CD103^+^ CD8^+^ T-cells. (A) Representative plots show expression of granzyme B by CD103-expressing CD8^+^ T-cells with (+DTx) or without (-DTx) treatment at 30 dpi. (B) Data presented show the percentage of granzyme B production by T_RM_ following peptide stimulation at the indicated time point. **p <0.001 DTx- versus DTx+ MCMV-infected at 30 dpi.

T_RM_ cells control reinfection and reactivation of infection because of their ability to produce potent recall responses, proliferate, and generate abundant effector functions to mediate cytotoxicity [16, 39]. We next sought to determine the effect to Treg depletion on granzyme B production, as a measure of potential cytotoxic activity, produced by T_RM_ cells. We assayed granzyme B production following peptide stimulation by brain lymphocytes obtained from DTx-treated and untreated animals at 30 dpi. Strikingly, CD103^+^CD8^+^ T-cells from Treg-sufficient animals demonstrated higher levels of granzyme B production when compared to those obtained from animals which lacked Tregs (Fig 11A). This difference was found to be significant: 15.9% of the CD103^+^CD8^+^T-cells produced granzyme B in Treg-sufficient animals, compared to 1.69% in Treg-deficient mice P<0.001 (Fig 11B).

## Discussion

Previous findings from our laboratory have demonstrated that depletion of Treg cells during acute MCMV infection leads to increased numbers of T lymphocytes within the brain, along with chronic reactive gliosis [8]. Results reported here provide evidence for increased proliferation of both CD8^+^ and CD4^+^ T-cell subsets within the brain following acute Treg depletion. By adopting a method of intravascular staining, we evaluated the proliferation status of lymphocytes present in capillaries which permeate the brain, as well as those present within the brain parenchyma. These findings revealed that lymphocytes proliferate in both the CNS microvasculature and brain parenchyma, and that Treg-depletion result in increased proliferation. The application of this intravascular staining protocol overcomes limitations of perfusion, which does not completely eliminate cells from the vasculature. This intravascular staining method has been reported to be useful in discriminating between cells that are truly tissue-localized from those present in the microvasculature, and its effectiveness has been well-demonstrated in tissue like lung and spleen [29, 31].

Given that Tregs are actively engaged in negative regulation of effector cells, we next assessed the activation state of the immune effectors. We found that CD8^+^ T-cells remained significantly activated from the acute phase of infection through the chronic phase in DTx-treated animals, when compared to untreated mice. Because of their potent and well-characterized anti-inflammatory effects, accumulation and retention of Tregs within the MCMV-infected brain could be beneficial in controlling overzealous, tissue damaging neuroimmune responses. Our laboratory has previously reported that brain infiltrating T-lymphocytes activate resident microglial cells through a mechanism involving IFN-γ [4]. So, we next analyzed the presence of proinflammatory cytokines through a kinetic time-course from acute through chronic phases of infection. Similar to observations made using other brain infection and stroke models, we observed increased production of IFN-γ and TNF-α following stimulation with a recognized T-cell epitope peptide by lymphocytes obtained from the brains of DTx-treated animals when compared to untreated mice [7, 40].

It is clear that Tregs are involved in variety of CNS diseases and stroke [41–43]. Thus, we also evaluated the proliferation and activation of the Treg cells themselves within MCMV-infected brains. Our data demonstrate increased proliferation and activation of Tregs as observed through Ki67 and ICOS expression, respectively, during the acute phase of infection (i.e. 7 dpi in DTx-untreated animals). In untreated, infected animals proliferation of Tregs decreased with progression of infection. An increase in Treg number as infection progresses has previously been reported during viral infection [7, 8], thus indicating that Treg proliferation decreases once their required numbers are reached. In addition, we also showed that Tregs become activated during acute infection and activation marker expression decreases through the chronic phase in Treg-sufficient animals. However, when we compare activation and proliferation of Tregs at 30 dpi in DTx-treated animals, where the Tregs rebounded by 14–20 dpi, we observed that their proliferation significantly increased in Treg-deficient animals in comparison to Treg-sufficient animals. Similar observations were also made regarding the activation of Tregs at 30 dpi, indicating, that cells which repopulate the infected brain may be functionally compromised. These finding were similar to our recently study which reported the inability of recovered Treg to suppress chronic reactive gliosis[8].

In recent years the concept that Tregs limit the extent of virus-induced immunopathology has been appreciated using several viral infection models, where tissue damage may not be a direct consequence of the virus itself [44–47]. Naïve CD8^+^ T-cells rapidly proliferate and differentiate into short-lived effector cells and long-lived memory cells following viral infection. An important subset of memory cells has been defined over the past decade, termed T_RM_ cells. These cells remain resident within extralymphoid tissues following immune responses and provide rapid protective immunity against previously encountered pathogens [14, 18, 35]. In this study, we investigated the role of Tregs in regulating the transition of the effector state to the generation of long-lived memory cells, as well as retention of these T_RM_ within the MCMV-infected brain. We first demonstrated the *ex-vivo* heterogeneity in expression of KLRG1 that marks the distinction between short-lived effector cells and long-lived memory cells. We noted in the brain of Treg-sufficient animals there are high expression levels of KLRG1 during the effector stage (7 dpi), which progressively decrease by 30 dpi_._ Expression of CD103, a marker for tissue resident memory cells in the CNS, increases at later time points (14 & 30 dpi). In contrast, in DTx-treated animals, KLRG1 expression was still evident by 30 dpi whereas expression of CD103 was significantly reduced at this time point, indicating that Treg-deficient mice had more effector cells and that the generation of resident-memory cells was impaired. Similar observations have been reported by Graham et.al.[7], where generation of T_RM_ cells was found to be impaired in Treg-deficient mice upon WNV infection. However, Graham et.al. did not find any difference in memory precursor cell (CD127^+^KLRG1^-^) levels in WNV-infected brain of DTx-treated and untreated animals at d 8 and 11 pi.

Since previous reports state that T_RM_ are strictly localized to nonlymphoid tissue, retention may be linked to the presence of TGF-β in those microenvironments. This cytokine is considered to be the driving force for expression of CD103 [7, 14, 39, 48]. Unlike the situation reported by Wakim et. al. [38], where VSV Ag persists in the brain up to 3 months and may drive CD103 expression, in our model lack of detectable Ag led us to speculate that CD103 expression on T_RM_ cells may be mediated through TGF-β production by Treg cells. Our results are indicative of the fact that heterogeneity in the expression of KLRG1 and CD127 following MCMV infection may be associated with the generation of two distinct populations of short-lived effector and long-lived memory cells during progression of infection. Thus, Treg cells appear to play a major role in the retention of T_RM_ cells within the brain following MCMV infection.

Findings from our study demonstrate that depletion of Tregs leads to activation of various immune cell populations within the brain, which persist out to at least 30 dpi. Elevated and prolonged immune responses to acute viral infection can be detrimental to brain cells and may lead to long-term neurological deficits. Treg cells isolated from the brain downregulate ongoing CNS inflammation following viral infection. Depletion of Treg cells (i.e. loss of their suppressive function) is not only linked with a hyperinflamed CNS, but it is also associated with decreased retention/development of T_RM_ cells. In addition, our laboratory has shown that Treg depletion is also associated with persistent chronic reactive gliosis, which leads to long-term neurological deficit in spatial learning and memory, thus emphasizing the important role of Tregs in maintaining parenchymal immune homeostasis.

T_RM_ cells are not only uniquely positioned within tissues, but also display rapid proliferation and immediate effector responses upon re-exposure to Ag [49]. In response to MCMV brain infection, CD8^+^ T-cells acquire effector functions which are essential for clearance of virus [12]. We next examined the effector functions of T_RM_ cells by investigating granzyme B production by these cells from DTx-treated versus untreated animals upon peptide restimulation. CD8^+^ CD103^+^ T-cells from Treg-sufficient animals, (i.e., the group which had more T_RM_ cells) showed higher production granzyme B in response to peptide re-stimulation. In contrast, T_RM_ cells from Dtx-treated animals showed decreased effector function as noted by granzyme B production. Likewise Wakim et. al., using a VSV brain infection model, demonstrated that CD103^+^ T_RM_ express high amount of granzyme B as opposed to CD103^-^ memory T-cells [38]. A similar observation was made by Casey et. al., using gut and intestinal T_RM_ that were primed with LCMV [50]. Thus, increased expression of effector molecules like granzyme B may assist T_RM_ to limit the spread of pathogen in immune privileged site like brain and may also help to enhance protection against viral spread following reactivation of latent infection.

Taken together, Tregs infiltrate the MCMV-infected brain where they regulate proliferation and activation of effector CD8^+^ and CD4^+^ T-cells. They also play crucial roles in the accumulation of T_RM_ cells within the brain. Results presented here emphasize the significance of these cells in maintaining the delicate balance between generation of effective immune responses against viral brain infection and immunopathogenic responses.

## Supporting Information

S1 Fig Acute DTx treatment of wild-type animals without the DTR has no impact on neuroinflammation. Wild-type C57/B6 mice were given acute DTx treatment as used in this study and described in (Fig 1A). Single cell suspensions of brain tissue obtained from infected C57/B6 mice (3animals/group with and without DTx) were collected and stained for flow cytometry with PE-Cy5-conjugated Abs specific for CD45, APC-labeled for CD8,e-F 450-labeled for CD4,PE-Cy7-labeled for KLRG1, PE-labeled for CD103 and Ki67 FITC–conjugated Abs. (A) Representative plot shows the CD45^hi^population which were further identified as CD8^+^and CD4^+^ T-cells. (B) Contour plots show proliferation frequency of CD8^+^T-cells from infected, untreated (-DTx) and DTx-treated (+DTx) animals at 14dpi. (C) Contour plots show proliferation frequency of CD4^+^ T-cells from infected, untreated (-DTx) and DTx-treated (+DTx) animals at 14dpi. (D) Contour plots shows T_**RM**_ (i.e., CD103^+^) cells gated on CD8^+^ T-cells from infected, untreated (-DTx) and DTx-treated (+DTx) animals at 14dpi. (E) Detection of MCMV IE1 transcripts within infected brains at 7, 14, &30 dpi.
(TIF)

S2 Fig CD8^+^ T-lymphocyte proliferation in cervical lymph nodes of infected mice following Treg depletion. Single cell suspensions of cervical lymph nodes obtained from infected Foxp3-DTR transgenic mice were collected and stained for flow cytometry with PE-Cy5-conjugated Abs specific for CD45, PE-Cy7-labeled for CD8, and Ki67 FITC–conjugated Abs. Contour plots show the proliferation frequency of CD8^+^ T-cells from infected, untreated (-DTx) versus DTx-treated (+DTx) animals at the indicated time points.
(TIF)

S3 Fig Increased proliferation of CD4^+^ T-lymphocytes in the cervical lymph nodes of infected mice following Treg depletion. Lymph node cells from MCMV-infected, Foxp3-DTR transgenic mice were collected at 7, 14, and 30 dpi. Cells were stained for flow cytometry analysis with PE-Cy5-conjugated Abs specific for CD45, e-F- 450-labeled for CD4, and Ki67 FITC–conjugated Abs. Contour plots show the proliferation frequency of CD4^+^ T-cells from infected, untreated (-DTx) and DTx-treated (+DTx) animals at the indicated time points.
(TIF)

S4 Fig Expression of CD127^+^ on T-cells within the infected brain following Treg depletion. CNS-derived mononuclear cells were gated on CD8^+^ T-cells and analyzed for expression of CD127 (a marker for memory T-cells). Pooled data show the percentage of CD8^+^ T-cells expressing CD127 at the indicated time points.
(TIF)

S5 Fig Expression of CD103^+^ T-cells in the cervical lymph nodes of infected mice following Treg depletion. Lymph node cells from MCMV-infected, Foxp3-DTR transgenic mice were collected at 7, 14, and 30 dpi. Cells were stained for flow cytometry analysis of T_**RM**_ cells. Representative contour plots show the frequency of KLRG1^+^ and CD103^+^cells (gated on CD8^+^T-cells)as well as the expression of CD103^+^cells onCD8^+^T-lymphocytesfrom infected, untreated (-DTx) and DTx-treated (+DTx) animals at 30dpi (upper panel & lower panel, respectively).
(TIF)
