# Supplementary figures and images for: Tregs Modulate Lymphocyte Proliferation, Activation, and Resident-Memory T-Cell Accumulation within the Brain during MCMV Infection
Source: PLoS One. 2015 Dec 31;10(12):e0145457. doi: 10.1371/journal.pone.0145457 (PMC4697843; doi:10.1371/journal.pone.0145457)

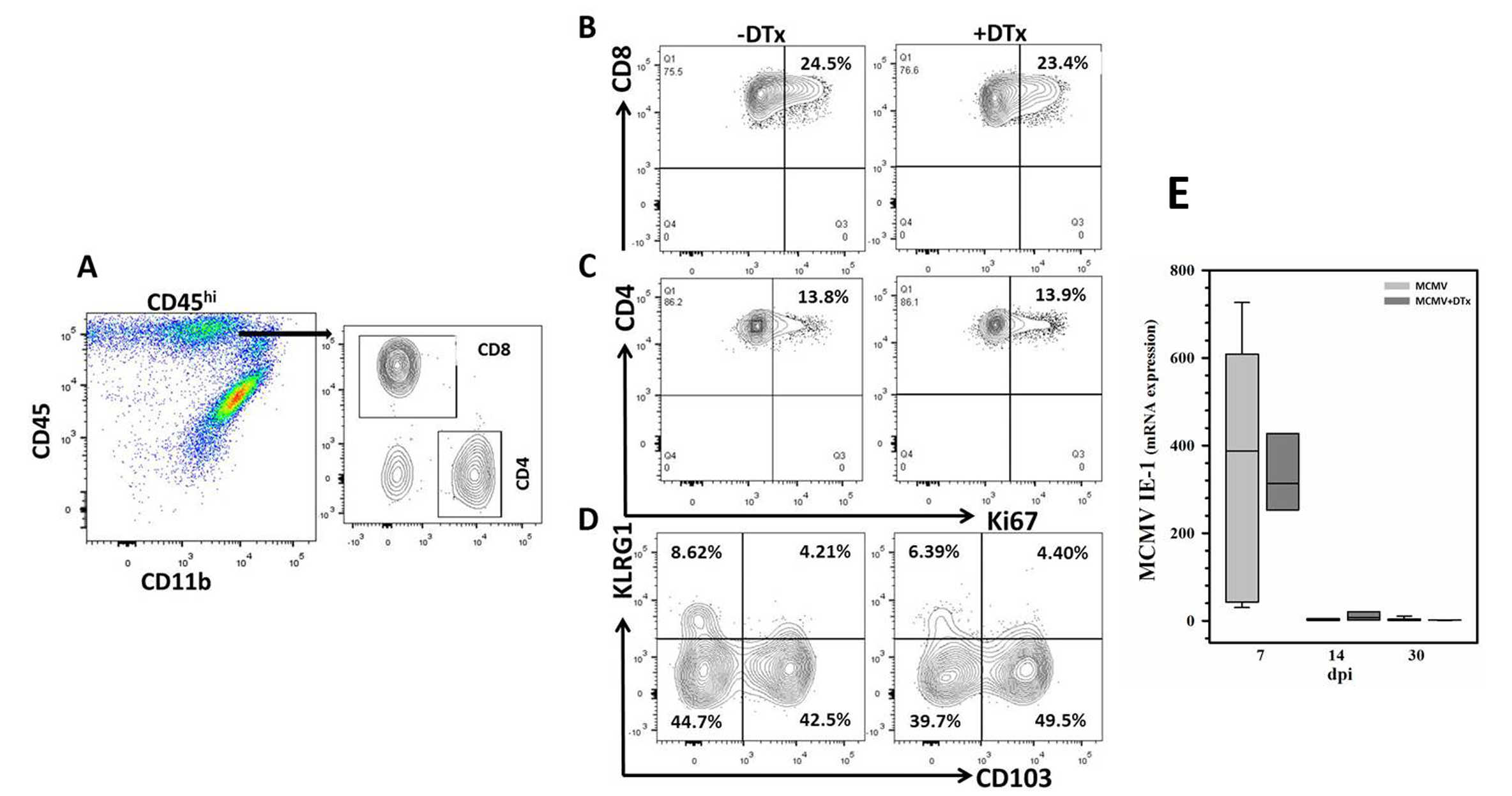

Supplement: S1 Fig — Wild-type C57/B6 mice were given acute DTx treatment as used in this study and described in (Fig 1A). Single cell suspensions of brain tissue obtained from infected C57/B6 mice (3animals/group with and without DTx) were collected and stained for flow cytometry with PE-Cy5-conjugated Abs specific for CD45, APC-labeled for CD8,e-F 450-labeled for CD4,PE-Cy7-labeled for KLRG1, PE-labeled for CD103 and Ki67 FITC–conjugated Abs. (A) Representative plot shows the CD45hipopulation which were further identified as CD8+and CD4+ T-cells. (B) Contour plots show proliferation frequency of CD8+T-cells from infected, untreated (-DTx) and DTx-treated (+DTx) animals at 14dpi. (C) Contour plots show proliferation frequency of CD4+ T-cells from infected, untreated (-DTx) and DTx-treated (+DTx) animals at 14dpi. (D) Contour plots shows TRM (i.e., CD103+) cells gated on CD8+ T-cells from infected, untreated (-DTx) and DTx-treated (+DTx) animals at 14dpi. (E) Detection of MCMV IE1 transcripts within infected brains at 7, 14, &30 dpi. (TIF) [file pone.0145457.s001.tif]

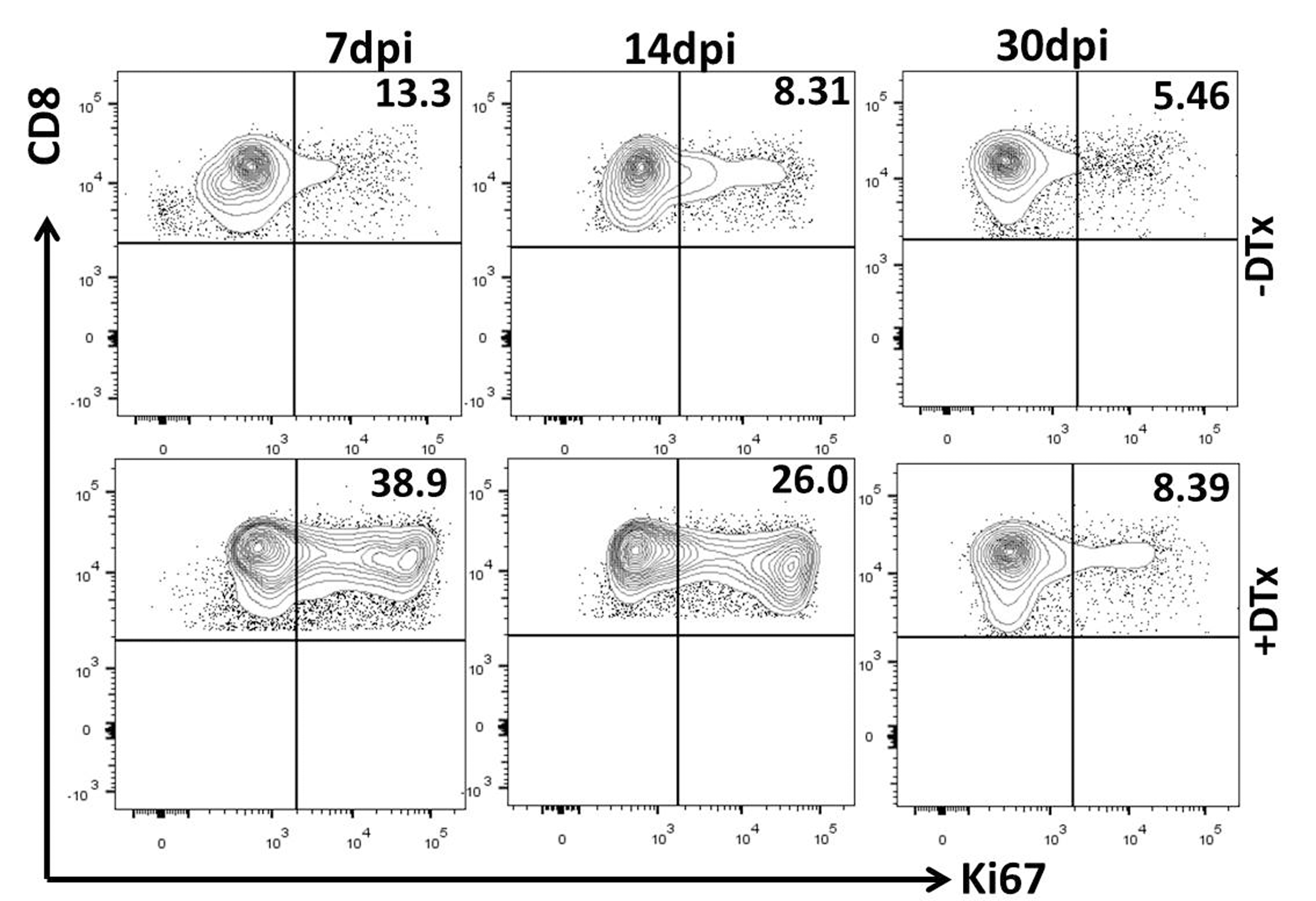

Supplement: S2 Fig — Single cell suspensions of cervical lymph nodes obtained from infected Foxp3-DTR transgenic mice were collected and stained for flow cytometry with PE-Cy5-conjugated Abs specific for CD45, PE-Cy7-labeled for CD8, and Ki67 FITC–conjugated Abs. Contour plots show the proliferation frequency of CD8+ T-cells from infected, untreated (-DTx) versus DTx-treated (+DTx) animals at the indicated time points. (TIF) [file pone.0145457.s002.tif]

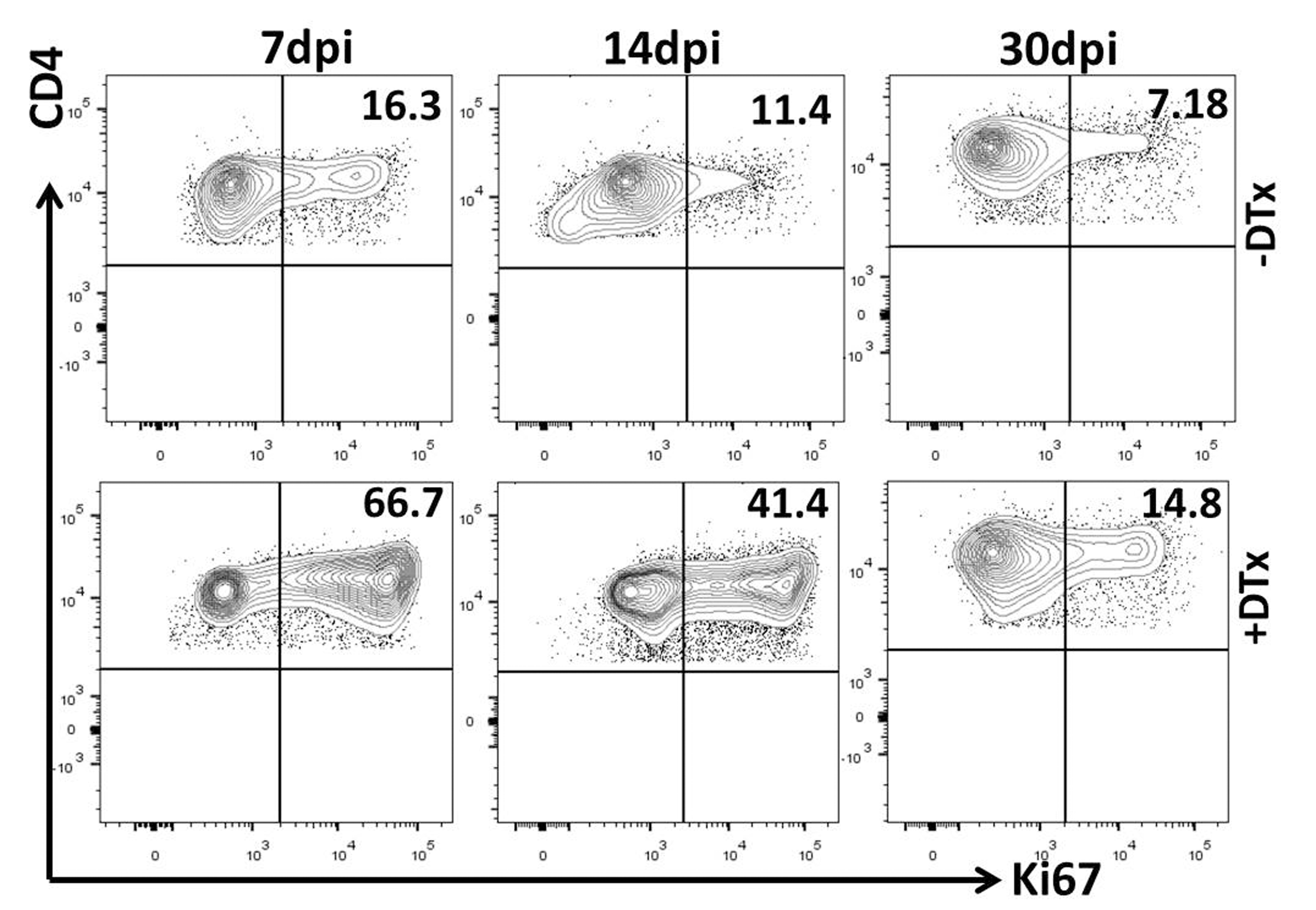

Supplement: S3 Fig — Lymph node cells from MCMV-infected, Foxp3-DTR transgenic mice were collected at 7, 14, and 30 dpi. Cells were stained for flow cytometry analysis with PE-Cy5-conjugated Abs specific for CD45, e-F- 450-labeled for CD4, and Ki67 FITC–conjugated Abs. Contour plots show the proliferation frequency of CD4+ T-cells from infected, untreated (-DTx) and DTx-treated (+DTx) animals at the indicated time points. (TIF) [file pone.0145457.s003.tif]

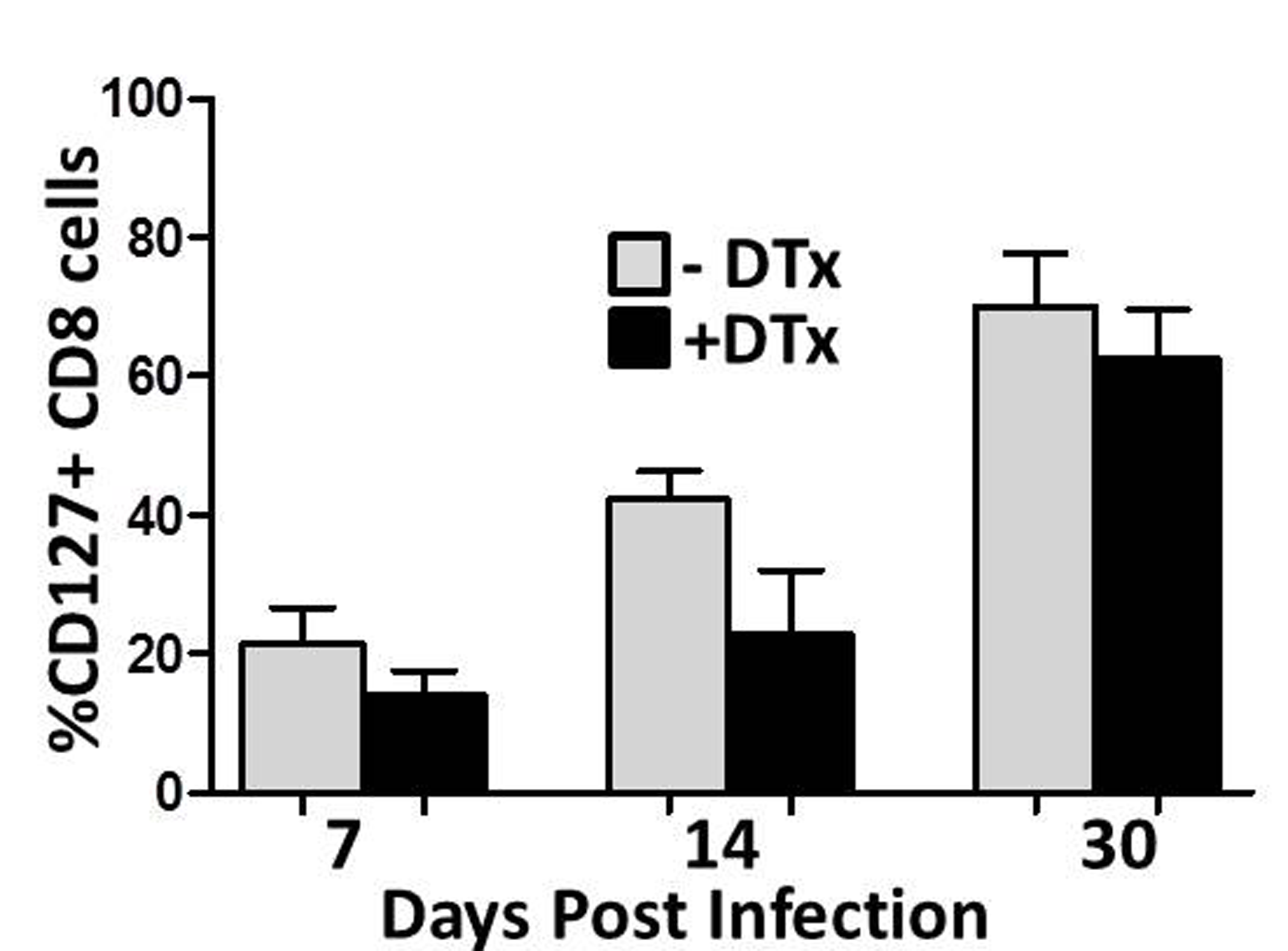

Supplement: S4 Fig — CNS-derived mononuclear cells were gated on CD8+ T-cells and analyzed for expression of CD127 (a marker for memory T-cells). Pooled data show the percentage of CD8+ T-cells expressing CD127 at the indicated time points. (TIF) [file pone.0145457.s004.tif]

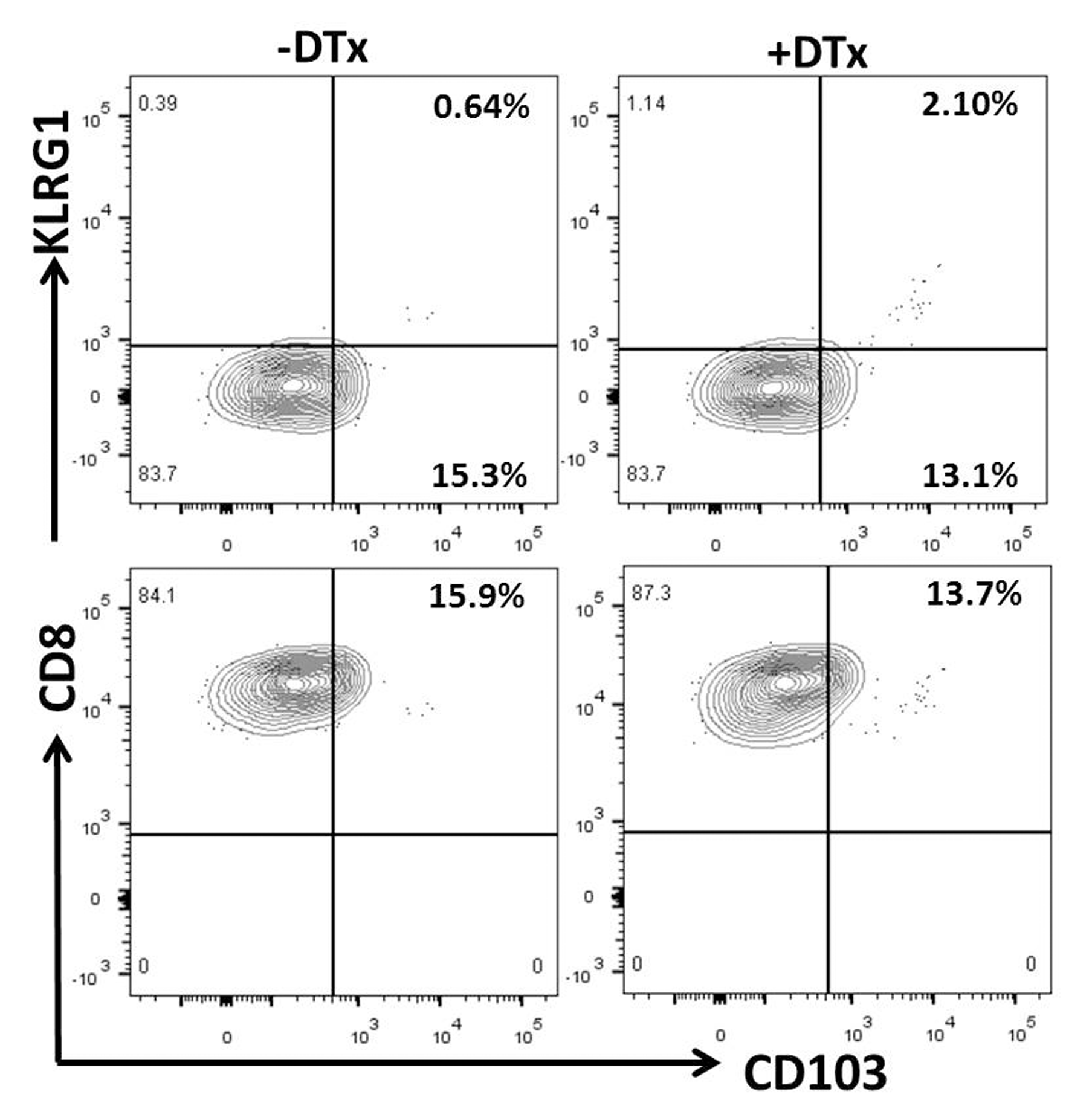

Supplement: S5 Fig — Lymph node cells from MCMV-infected, Foxp3-DTR transgenic mice were collected at 7, 14, and 30 dpi. Cells were stained for flow cytometry analysis of TRM cells. Representative contour plots show the frequency of KLRG1+ and CD103+cells (gated on CD8+T-cells)as well as the expression of CD103+cells onCD8+T-lymphocytesfrom infected, untreated (-DTx) and DTx-treated (+DTx) animals at 30dpi (upper panel & lower panel, respectively). (TIF) [file pone.0145457.s005.tif]
